# Cultural ecosystem services in European grasslands: A systematic review of threats

**DOI:** 10.1007/s13280-022-01755-7

**Published:** 2022-07-06

**Authors:** Raoul Pellaton, Eszter Lellei-Kovács, András Báldi

**Affiliations:** Lendület Ecosystem Services Research Group, Institute of Ecology and Botany, Centre for Ecological Research, Alkotmány út 2-4, Vácrátót, 2163 Hungary

**Keywords:** Direct drivers, Nature conservation, Permanent grassland, Threat mitigation, Tourism

## Abstract

The degradation of ecosystems threatens the provision of ecosystem services and limits human well-being. This systematic literature review evaluates the threats surrounding cultural ecosystem services (CES), namely recreation and landscape aesthetics in European permanent grasslands. We identified underlying causes, direct threats, consequences and suggested solutions for threat mitigation. The most common threats were land-use and management change processes, followed by social attitude, industrial developments and natural threats. However, recreational activities also created negative feedback, affecting the ecosystem, biodiversity and CES, most frequently in the form of various touristic activities. Suggested solutions were most commonly socio-economic and institutional measures to enhance rural communities, as well as improving communication with relevant stakeholders. CES play a crucial role in reconnecting people with nature, and their consequent acknowledgement and incorporation into future ecosystem service frameworks and agri-environmental policy developments are key elements in supporting future sustainable grassland management.

**Supplementary Information:**

The online version contains supplementary material available at 10.1007/s13280-022-01755-7.

## Introduction

Humans are facing significant challenges due to the degradation of ecosystems, a trend exacerbated by climate change that continued even during the COVID-19 pandemic (IPBES 2018; McNeely 2021). An understanding of the degradation and other processes that threaten diverse ecosystem services is urgently needed to maintain human well-being (Kosanic and Petzold 2020). While supporting, provisioning and regulating ecosystem services are long recognised and discussed, cultural ecosystem services (CES) have only recently become more prominent, shifting the focus from an economics-centred approach to a socio-ecological approach (Plieninger et al. 2015a). In the Millennium Ecosystem Assessment (MEA 2005a), CES were defined as the non-material benefits for people from recreational and aesthetic experience, including spiritual and educational values (see also the Common International Classification of Ecosystem Services (CICES) in Haines-Young and Potschin 2018).

Permanent grasslands are an important land use in Europe, covering about 812 160 km^2^ (40%) of the agricultural area (Faostat 2021) and harbouring valuable and rich biodiversity (WallisDeVries et al. 2002; Feurdean et al. 2018). Permanent grasslands consist of land where grasses and other herbaceous plants grow naturally or were sown but have not been included in a crop rotation for at least 5 years (EC 2011). Many permanent grasslands are intensively managed as productive grasslands, through grazing, mowing, manufactured fertiliser application or reseeding, while others are managed more extensively. They cover a wide range of grassland types, from desertic to mesic types, and include Alpine grasslands, dehesas and the Pannonian steppe (Silva et al. 2008; IPBES 2018; Tonn et al. 2020).

European stakeholders consider feed for livestock to be the main provisioning service of grasslands (van den Pol-van Dasselaar et al. 2013). Among all the ecosystem services, cultural services are ranked last (van den Pol-van Dasselaar et al. 2013; Zhao et al. 2020). The multiple ecosystem services of permanent grasslands are often in a trade-off relation with each other (Zhao et al. 2020). For example, grassland’s recreational and aesthetic qualities are often negatively correlated with land-use intensity (Raudsepp-Hearne et al. 2010; Allan et al. 2015). CES may be at risk with an increasing demand for profitable agriculture (Allan et al. 2015). CES are often difficult to measure and understand, thus tend to be ignored (Chan et al. 2012), undervalued (Rodríguez et al. 2006), neglected (Chan et al. 2012; Tengberg et al. 2012), and data deficient (DeFries et al. 2005; Rodríguez et al. 2006). They cover multiple disciplines and require collaboration between social and natural scientists (Hernández-Morcillo et al. 2013) and the inclusion of traditional knowledge of indigenous people and local communities (Díaz et al. 2018; Hill et al. 2020).

Literature on CES is still not abundant compared to provisioning or regulating ecosystem services (Hirons et al. 2016; Zhao et al. 2020; Schils et al. 2022). Although CES are increasingly identified as essential services (Daniel et al. 2012; Milcu et al. 2013), they are still underrepresented in global initiatives such as the Sustainable Development Goals or the Aichi Targets (Daniel et al. 2012; Hirons et al. 2016; Geijzendorffer et al. 2017). Yet, CES can serve as the “binding elements between social and ecological conceptual constructs”, which is a fundamental concept for sustainability (Milcu et al. 2013: 10). Studies on CES aim to explore perceptions, beliefs and identities and to raise awareness of the need for ecosystem protection (Chan et al. 2012; Daniel et al. 2012). Two of the most frequently studied CES are landscape aesthetics and recreation (including the various forms of tourism), as they can be assessed using a monetary approach; the common currency for ecosystem service studies (Milcu et al. 2013; Fish et al. 2016; Zhao et al. 2020).

Previous scientific reviews on CES were, to the authors’ knowledge, not fully dedicated to the analysis of threats (see e.g. Milcu et al. 2013; Plieninger et al. 2015a; Kosanic and Petzold 2020; Zhao et al. 2020), especially not in permanent grassland systems. Yet, the very existence of threats due to human expansion is what first brought increased attention to aesthetic ecosystem services (de Groot et al. 2005). In order to understand and mitigate their effect on human well-being and the environment, processes that threaten and negatively impact CES and well-being should be given greater emphasis in the literature (Hirons et al. 2016). Due to their high relevance to human well-being, we included landscape aesthetics and recreation in our analysis (Milcu et al. 2013; Zhao et al. 2020).

### Conceptual framework

**Fig. 1:**
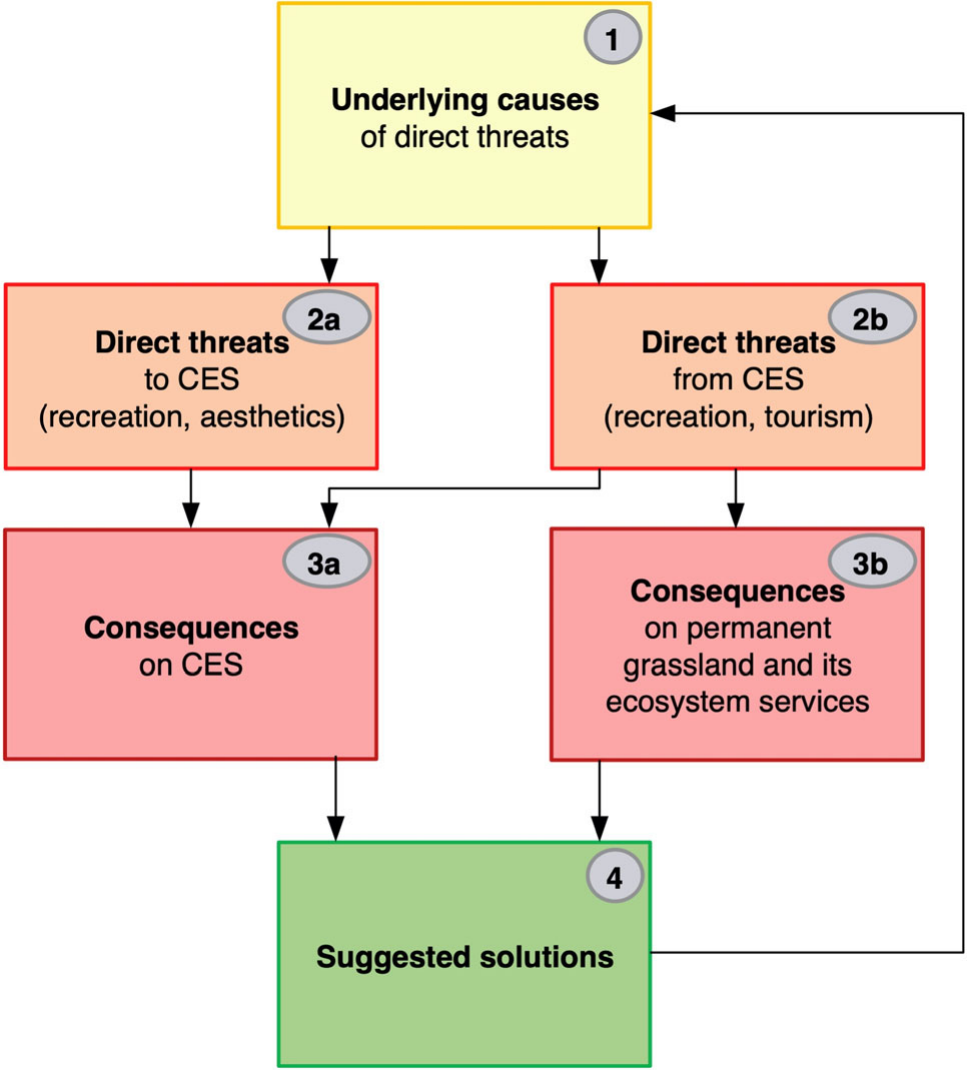
The conceptual framework of presumed interactions regarding threats surrounding the cultural ecosystem services (CES) of permanent grasslands in Europe. Underlying causes [1] can be institutional, economic, climatic, or social factors triggering alterations in grasslands’ general conditions and services. The ecosystem may be directly exposed to various changes (i.e. direct threats) to different grassland functions related to CES or other functions of the grassland. Two aspects of threats were considered: first, threats to CES [2a] that negatively affect the CES of permanent grasslands, and second, threats from CES [2b], namely negative impacts of CES on biodiversity, ecosystems and their ecosystem services. Consequences of these direct threats may affect CES [3a] or the ecosystem (i.e. permanent grasslands) and its ecosystem services [3b]. Suggested solutions [4] to mitigate threats and to ensure the continued supply of CES in permanent grasslands reflect back to the underlying causes and may help reduce those threats. Numbers in the boxes refer to the research questions, colours to those in Figs. 2 and 3

Based on our systematic review, we created a conceptual framework for discussing and better understanding the threats around CES (Fig. 1). In this framework, we distinguish four levels: Underlying causes, direct threats, consequences and suggested solutions. Threats affecting grasslands and their CES can be direct or indirect (for the exact definition, see Díaz et al. 2015; IPBES 2018). Therefore, we distinguish in our framework between *underlying causes* (Geist and Lambin 2002; Plieninger et al. 2016) on the first level (see Fig. 1, note the numbers in the boxes) and *direct threats* (Salafsky et al. 2008) around the CES of permanent grasslands on the second level (for a justification of this terminology, see methods section). Threats not only impact beneficial ecosystem services but may also be caused by their usage (Shapiro and Báldi 2014). In our framework, we therefore differentiate between threats affecting CES (“direct threats to CES”) and threats from CES that affect grassland ecosystems and their services (“direct threats from CES”). For example, the increasing demand for recreation in recent decades poses several risks to the environment (Bhattacharya et al. 2005; de Groot et al. 2005). Therefore, the second level (i.e. direct threats) of our framework is split into two parts: one is “Direct threats to CES”, and the other is “Direct threats from CES”. Threats may result in diverse negative responses (“consequences''), hampering the functionality of grasslands in providing services, changing not only the CES performance but also potentially leading to the destruction of the grassland ecosystem. In our framework, we differentiate between consequences on the grassland ecosystem and its services, and consequences purely on CES. Mitigation measures (“suggested solutions”) may be introduced at any level of the threat cascade, indicating what changes to the underlying factors could reduce the specific threats to the ecosystem or its services (including CES).

In this systematic review, we aim to evaluate what threats to recreation and landscape aesthetics have been described in the literature, what consequences they may have and what solutions have been suggested for their mitigation. Considering the diverse types of threats, we formulated the following research questions (numbers correspond with those in Fig. 1).

- What are the underlying causes of threats to or from cultural ecosystem services (CES, i.e. recreation and landscape aesthetics) provided by European permanent grasslands?
- What are the most common direct threats to the CES provided by European permanent grasslands?
- What are the most common threats to European grasslands and the ecosystem services generated by grassland-associated recreational activities and tourism?
- What are the consequences of threats to CES in European permanent grasslands?
- What are the consequences of threats from CES to European permanent grasslands and feedback to their cultural ecosystem services?
- What suggested solutions are available to address these aspects and mitigate the identified threats?

## Methods

### Data compilation

As part of the SUPER-G project (developing SUstainable PERmanent Grassland systems and policies; https://www.super-g.eu), the Scopus and CAB abstract databases were searched on 5 November 2019 for grassland CES in Europe (for a detailed description of the search, selection method and extraction, see Schils et al. 2022; with key points mentioned below). Basic search criteria were publication after 1980 in the English language. The search string included the word “grass” and related synonyms, as well as relevant terms regarding landscape aesthetics and recreation (see Table S1). The resulting 17 979 papers were uploaded into the EPPI reviewer 4 tool (http://eppi.ioe.ac.uk/cms), a systematic review analysis software. Finally, after removing duplicates, 13 719 articles remained for screening.

### Paper selection and data extraction

As a first step, titles and abstracts were screened on relevance and filtered based on a set of exclusion criteria (see Supplementary Appendix S1). In the first round of screening, 13 523 papers were excluded (see Fig. S1). As a second step, the full texts of the remaining 196 papers were scanned, of which 71 papers were selected that contained at least one of the following aspects:

- threats to CES and permanent grasslands,
- threats to grasslands from CES,
- solutions to prevent or reverse threats.

The selected 71 papers (see the complete list of included publications in Appendix S2) were read, and data were extracted into a data extraction form (Table S2a/b). The selected papers contained 77 studies, with four papers including two or more studies from different countries, which were analysed separately. The elicited data included bibliographical information, country and region of the study, spatial scale (i.e. country, regional, landscape or plot scale), grassland type, threat type, underlying causes and consequences and suggested solutions. The paper selection was made independently by the two first authors, and in divergent cases, a consensus was developed.

### Data analysis

The selected 77 studies were analysed qualitatively. CES are generally assessed qualitatively, which differentiates them from other ecosystem services (Milcu et al. 2013). A substantial part of the data analysis was the identification and classification of threats and their respective underlying causes and consequences (Supplementary Table S3; see also Bürgi et al. 2005). Furthermore, we analysed what solutions had been suggested to prevent or mitigate negative effects. Data were summarised, classified and compared in two main categories: first, threats to CES, namely factors that negatively impact the CES of permanent grasslands, and second, threats from CES, namely negative impacts of CES on the ecosystem and its other ecosystem services (see also Fig. 1).

As no classification scheme fully covered the types of threats we encountered regarding CES of permanent grasslands (see Table S3), we compared our findings on direct threats to the *direct threats classification on biodiversity* by the IUCN-Conservation Measures Partnership (IUCN-CMP 2019; first described by Salafsky et al. 2008) and underlying causes to the *IPBES categorisation of indirect drivers* (IPBES 2018), which both serve as international standards. Although originally developed for biodiversity and the assessment of species and habitats (IUCN Red Lists; IUCN-CMP 2019), these classification systems served as a good base for elaborating a classification system of the threat types to and from CES that we found in the reviewed literature at the respective levels (i.e. underlying causes and direct threats). Although the IUCN-CMP classification is largely congruent with the IPBES categorisation of direct drivers (IPBES 2018, 2019), its advantage is the category of “recreational activities” that captures the small-scale impact of recreation on the habitat. This classifying step was done independently by the two first authors, and a consensus was developed.

IPBES (2018) distinguishes institutional, demographic, economic, scientific and cultural indirect drivers (i.e. underlying factors), to which we added the climatic driver as it has an indirect impact on touristic activities (see also Plieninger et al. 2016). Additionally, we split the economic driver into socio-economic, institutional and purely economic factors, as they were considered distinct categories. The socio-economic driver contains economic factors where the social environment is in close interaction with the economic processes. For the “Threats from CES” analysis, the demand for recreation was introduced as an additional underlying factor, a category similar to "Lifestyle, consumption" according to the IPBES classification (IPBES 2018), although that did not fully cover the scope of this systematic review. For the direct threats, a large category not captured by either classification system was social attitudes, such as the disapproval of nature conservation measures, indifference towards the development of recreational services and the lack of appreciation for existing cultural values. Furthermore, both the absence and overwhelming presence of infrastructure are potential direct threats to the livelihoods of rural areas, although for different reasons (see “Results”). Consequences and suggested solutions were classified in broad categories to cover the variety of mentioned threats. We developed them post-hoc, as no classification existed that proposed such categories. The categories of the suggested solutions were created based on those in the underlying causes to reflect whether they were of socio-economic, institutional, infrastructural, demographic or of other nature.

During the evaluation process, the number of underlying causes, threats, consequences and solutions was counted within each of the 77 studies. Consequently, the same category may appear several times for a single study if mentioned in a different context or if related to another threat category.

## Results

### Threats to CES (recreation, aesthetics)

**Fig. 2:**
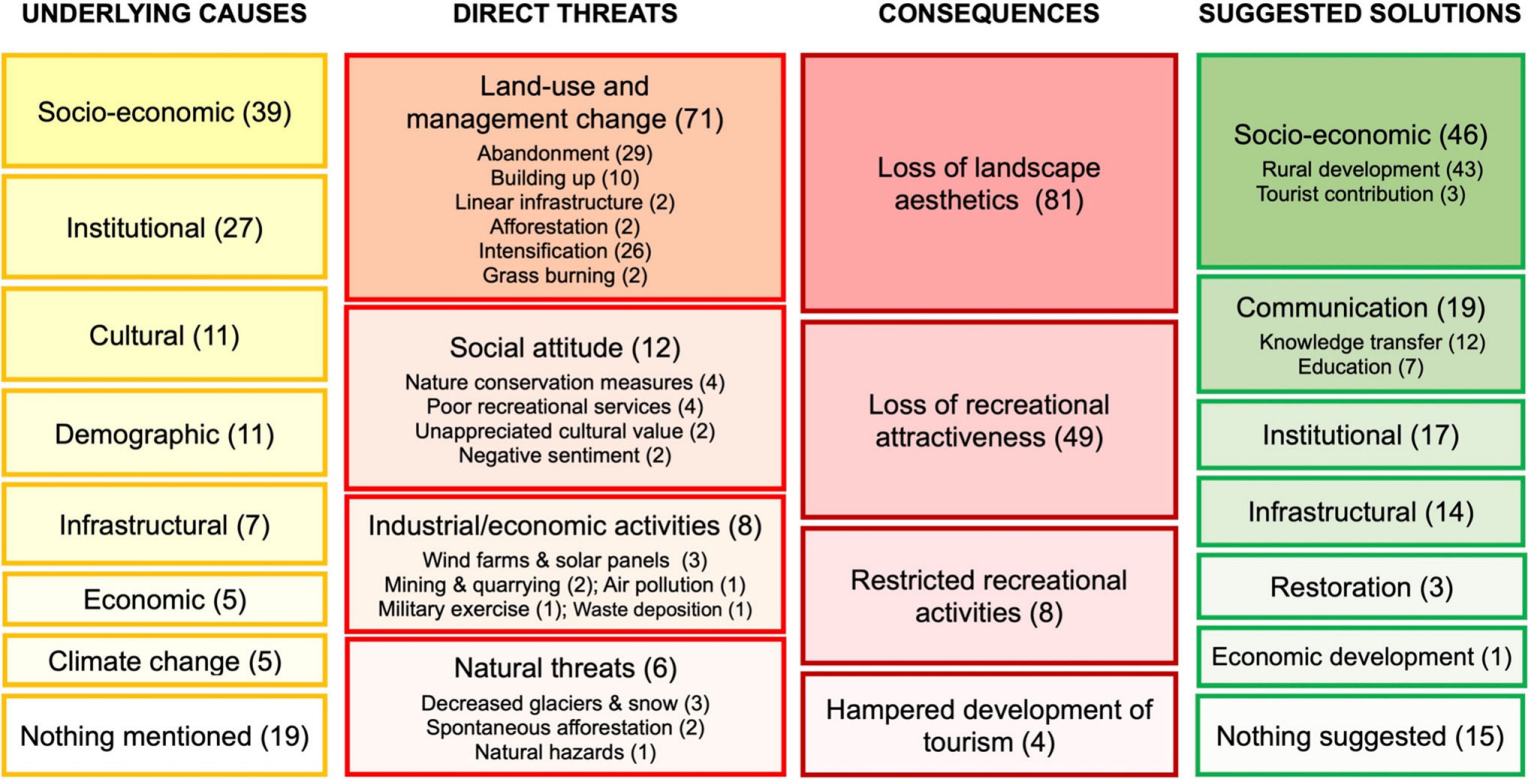
Threats to cultural ecosystem services in permanent grasslands based on the conceptual framework (Fig. 1). The number of studies involved is mentioned in parentheses. Note that a study can contribute to more than one box in a column if several aspects were mentioned. For a detailed representation with links between the boxes, see Supplementary Fig. S5

Fifty-one studies taken from 47 papers on threatened CES were included in the analysis (for an introductory outline of the results see Appendix S3). Following our conceptual framework (Fig. 1), we summarised the underlying causes, direct threats, consequences and suggested solutions mentioned in the extracted studies (Fig. 2; see also Supplementary Fig. S4).

#### Underlying causes

Based on the IPBES classification (IPBES 2018), seven types of underlying causes were mentioned in the reviewed studies (see Fig. 2 and Supplementary Table S4), of which socio-economic factors had the highest frequency (39 cases). They included economic aspects related to a lack of profitability and motivation of the local population to continue farming, as well as detrimental market forces, for example, against local products that can reduce income from farming. The second-most frequent underlying cause (27 cases) were institutional aspects, including strict regulations controlling recreational activities and other related regulations (e.g. privatisation, urban development) that do not support CES. In other cases, the lack of regulations led to land speculation and the related conversion of grasslands into built-up areas. In close connection with pure economics, the loss of governmental financial support led to a decrease in grassland areas, as they were abandoned. The loss of culture and traditions (11 cases) was commonly mentioned together with socio-economic changes. However, culture also had a controversial role, for example, when locals avoided a nature reserve due to negative memories or when there were conflicts between different groups about their perceptions of aesthetic landscapes. Demographic factors (11 cases) included the emigration of young people and an ageing population, leading to an unbalanced age structure. The related disrupted continuity of farming families led to the abandonment of traditional farming activities. On the other hand, urbanisation and the demand for residential buildings in grassland areas were caused by a growing population. Infrastructure (7 cases) was reported to be insufficient for tourism development and local access, restraining CES usage, or overabundant with roads supporting high traffic volumes in natural areas. Industrial and economic influences (5 cases) included large industrial or agricultural developments, the construction of renewable energy plants and technological changes. Finally, climate change (5 cases) was mentioned in the context of rising temperatures. In 19 cases, no underlying causes were mentioned targeting specific threats.

#### Direct threats

A total of 95 direct threats provided an insight into the diversity of threats affecting the function and CES of European grasslands. Based on the IUCN-CMP direct threat classification (IUCN-CMP 2019), four major groups of direct threats could be distinguished. The largest group was land-use and land management changes with 71 cases, distinguishing four land-use change and two land management change categories (see Fig. 2). Land abandonment was mostly associated with spontaneous overgrowth (27 out of 29 cases), an increase in homogeneous landscapes and a declining number of grazing animals and shepherds. Agricultural intensification (26 cases) included increasing inputs and stocking rates on grasslands and their conversion to arable land, pursuing a productivity-oriented agriculture on easily accessible land. It furthermore involved structural changes (fewer but larger farms and more animals on fewer farms), increased fertiliser use, and a shift of people’s valuation of importance from aesthetic and moral values to economic values. In 11 cases, abandonment and management intensification were mentioned as parallel processes. In 10 cases, built-up areas replacing grasslands was mentioned as a threat, either in the form of urbanisation (for both general and touristic purposes) or by establishing industrial features such as greenhouses or silos. A specific subcategory of built-up areas was the fencing off of private property following land tenure changes which eliminated recreational routes and restricted access for the public. Two papers mentioned linear infrastructures (highways and roads with high traffic volumes) as a source of disturbance and a hazard for tourists. A further two cases each dealt with grassland afforestation and grass burning.

The second-largest group of threats was related to social attitude (12 cases), a category with no equivalent in biodiversity science. Nature conservation measures were disapproved in four cases, as they were perceived as limiting recreational activities. Poor recreational services (4 cases) resulted from insufficient infrastructural development in the region, which could threaten recreation because of poor access to otherwise attractive places and a lack of local services. The lack of appreciation for cultural values posed a threat to a continued CES supply in two cases. In a further two studies, people had negative sentiments towards a place because of its history.

The third threat group incorporated industrial and economic activities (8 cases). In three studies, wind farms and solar panels impaired landscape aesthetics, and in two cases, mining and quarrying induced threats to both recreational activities and aesthetics through water-borne, air-borne and noise pollution. Industrial air pollution (1 case) caused both air pollution and aesthetic damage to the landscape. In one case each, military exercises and waste deposition were reported as threats to recreational activities and aesthetic values.

The fourth threat category was natural threats (6 cases), namely retreating glaciers and snow-deficient winters, spontaneous afforestation in place of melting glaciers and natural hazards in thawing permafrost areas. All the threats in this category affected both landscape aesthetics and recreational activities such as hiking.

#### Consequences

The reported consequences of these threats could be divided into two major groups: First, the reduced appeal of grasslands for recreation, and second, the decreased aesthetic value because of a homogenous landscape. Many threats affected recreation and aesthetics simultaneously. The loss of landscape aesthetics was the more dominant consequence with 81 cases, followed by reduced recreational appeal in 49 cases, restricted recreational activities in eight cases and hampered tourism development in four cases. Consequences of abandonment were the economic loss for the tourism industry and negative effects for hikers due to landscapes with reduced access for walking. It also had negative aesthetic effects through a reduction or a complete loss of panoramic views. In fact, both more intensive and too little management (abandonment) led to decreased cultural values (both aesthetic and recreational). The loss of aesthetic and recreational appeal, including hampered tourism development, lowered the frequency of tourist visits, while restrictions (due to nature protection or private property) to prevent access induced conflicts. They all led to tourists looking for other destinations, a reduced length of stay or a decreased willingness-to-pay (WTP) for local services. Yet, it was also mentioned that when places of high natural value are threatened, the exclusion of tourists may be necessary. In other situations, however, such strict regulations threatened the livelihood of farmers when their primary source of income was tourism or when management was no longer profitable, leading to further abandonment and the loss of CES.

#### Suggested solutions

Socio-economic solutions were by far the most widely suggested solution type (46 cases) and covered a wide range of mitigation measures for the development of rural areas. The solutions encompassed financial support from governments (e.g. agri-environmental payment), the stimulation of employment in rural areas, farm income diversification (e.g. the promotion of local food and products), improved market access, but also conservation measures and the development of sustainable rural tourism. All these approaches were reported in the reviewed literature to ensure local livelihoods, preserve a healthy environment and help to develop a sustainable, local socio-economic system for the given socio-economic context. The proper organisation of tourism may help to maintain traditional landscapes and activities. Communication was the second-most suggested solution (19 cases), including both transfer of knowledge and education. That covered various aspects, such as increased cooperation between stakeholders, their incorporation into landscape planning and decision-making, and enhanced valuation of CES. The education of locals and tourists was proposed to promote their understanding of the importance and functions of grassland ecosystems. Institutional solutions (17 cases) were typically regulations such as restricted industrial development in sensitive areas, adapted conservation strategies and governmental regulations ensuring sustainable maintenance and harmonising tourism and conservation. The suggested infrastructural investments (14 cases) included the development of tourism in response to environmental changes, with improved access to remote areas; the use of artificial snow at lower elevations or creating pistes at higher elevations; and the construction of new hiking trails and recreational ‘furniture’ (e.g. benches, direction signs). Finally, the restoration of the ecosystem functions (hydrology, connectivity, recreation) was suggested in three cases and purely economic development in one case. In 15 cases, no suggestions were made to reduce threats to CES.

### Threats from CES (recreation, tourism)

**Fig. 3:**
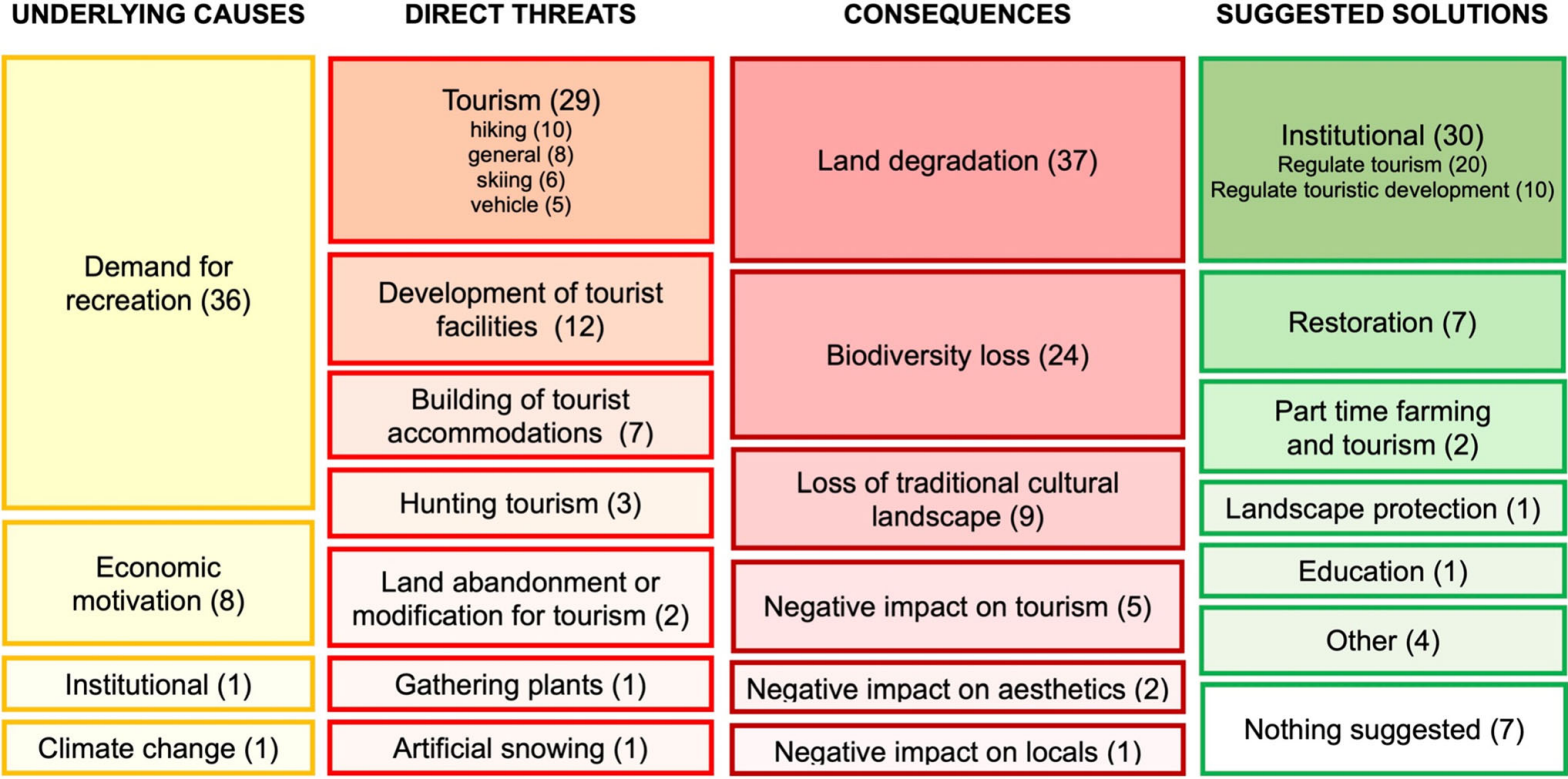
Cultural ecosystem services as threats to permanent grasslands based on the conceptual framework (Fig. 1). The number of involved studies is mentioned in parentheses. Note that a study can contribute to more than one box in a column if several aspects were mentioned. For a detailed representation with links between the boxes, see Supplementary Fig. S6

An overall 37 reviewed studies (in 36 papers) contained threats caused by CES (Fig. 3; see also Supplementary Fig. S5).

#### Underlying causes

The most frequently mentioned underlying cause for permanent grassland use was the demand for recreation (36 out of 37 selected studies). The second-most frequently mentioned underlying cause was economic factors (8 cases). All were related to tourism becoming more economically profitable than agriculture. Institutional backgrounds, such as defective regulations or lack of regulations, were discussed as the underlying cause of two threats. Climate change was mentioned once in the context of decreasing snow reliability, suggesting that the creation of artificial snow will be intensified to ensure the continued provision of CES.

#### Direct threats

Based on the IUCN-CMP direct threat classification (IUCN-CMP 2019), seven main threat categories could be distinguished. The most abundant threats were disturbances and pressures by tourism (29 cases), where four subcategories were differentiated, namely (1) general disturbances on ecosystems and people (e.g. high traffic frequency, diverse activities at the same place, etc.); (2) hiking and related trampling effects; (3) skiing, with similar effects like hiking, together with the disturbance of bird species in the surrounding areas; and (4) recreational vehicles for activities such as motorised sports (e.g. snowmobiling) or biking, where the physical impact was the main disturbance, together with noise and air pollution and impacts on wildlife. The second threat category was the development of tourist facilities (i.e. tourist infrastructure such as roads and ski lifts; 12 cases), followed by the building of accommodation for tourists (e.g. summer houses, hotels, mountain cabins), as a specific type of urban development competing with grasslands (7 cases). Minor threats were hunting tourism (3 cases) and gathering plant souvenirs (1 case) as sources of disturbance in grasslands. Abandonment of grassland management because of more lucrative jobs in tourism, land modification for recreational activities, and grassland eutrophication due to using artificial snow were each mentioned once.

#### Consequences

The most frequent consequences were land degradation processes (37 cases), such as reduced aboveground biomass, vegetation cover, species frequency and composition, and changes in the cellular structures of trampled plants. Furthermore, land degradation was connected to soil erosion and compaction, soil nutrient changes and sometimes even the total disappearance of grasslands due to buildings or recreational activities. Biodiversity loss was the second-most mentioned consequence (24 cases), often co-occurring with land degradation. Minor consequences were the loss of traditional cultural landscape (9 cases), the negative impact on tourism (5 cases) and aesthetic values (2 cases), and finally, the negative impact on residents of an increase in tourist numbers (1 case).

#### Suggested solutions

The regulation of tourism and economic development were suggested most often, using institutional and economic tools to avoid the overuse of the ecosystem (20 cases). Several reviewed studies suggested limiting visitors or implementing a frequentation management plan in sensitive areas or according to season to prevent negative impacts of human trampling. Alternatively, it was suggested to concentrate tourists on a few areas rather than let them spread or to monitor the negative effects with sensitive indicator species. The second-most frequently mentioned solutions were the regulation of urban development through land-use planning, conflict management and collaborations, among others (10 cases). Habitat restoration was noted in seven cases as an important tool to mitigate the negative impacts of CES. The unfavourable impacts of ski pistes were suggested to be restored by reseeding and mowing management. Infrequently suggested solutions were the development of an appropriate conservation strategy, the protection of the landscape, the increase of landscape multifunctionality, improved communication and education, the reconstruction of routes relieving sensitive natural areas and the diversification of income through a combination of part-time farming and working in tourism. Seven studies did not report any obvious solutions.

## Discussion

CES are generally not as well understood as most other ecosystem services (Chan et al. 2012; Schils et al. 2022), which is even more true for the various threats that imperil the continued supply of CES in permanent grasslands. In this systematic review, we provide an overview of such threats that have been described in the literature. The biggest direct threat to CES was land-use and land management change, mainly through abandonment and agricultural intensification. We also showed that human activities related to CES constitute a threat to permanent grasslands (see also IUCN-CMP 2019), primarily because of the negative impacts of tourist activities such as hiking or skiing. Suggested solutions to combat the various threats or their underlying causes and consequences mainly consisted of socio-economic interventions, using institutional and economic tools.

We further found evidence for the direct inter-linkage of threatened landscape aesthetics and recreation. Areas that were perceived as threatened in their aesthetics were also less appealing for recreation (see also Daniel et al. 2012). Such close connections and dependencies between CES stress their importance, but pose a challenge when studying them separately (de Groot et al. 2005; Daniel et al. 2012; EEA 2017).

### Threats to CES (recreation, aesthetics)

Land-use abandonment, agricultural intensification, urban development and other closely related negative impacts were the most dominant threats to CES, affecting both recreation and landscape aesthetics. These effects were previously identified as the most studied direct drivers of CES change (Milcu et al. 2013) yet were not extensively discussed. Here we provide clear evidence from the reviewed literature that land-use and land management change are the most commonly mentioned threats to CES in permanent grasslands.

The predominant drivers of land-use and land management change in the second half of the twentieth century in European permanent grasslands were the intensification of productive grasslands and the concurrent abandonment and encroachment of marginal areas (Stoate et al. 2009; Middleton 2013; Skokanová et al. 2016). Considerable areas of natural and semi-natural habitats have since been lost or were severely fragmented and have led to a substantial loss of related ecosystem services (EEA 2015), with CES being no exception. Reasons behind land-use and land management changes are primarily socio-economic, institutional or demographic (Rey Benayas et al. 2007). Abandonment is often the direct cause of cessation of management (Keenleyside and Tucker 2010; Beilin et al. 2014; Skokanová et al. 2016) and especially traditional management (MacDonald et al. 2000; Peco et al. 2006). The reasons for abandonment found in this systematic review were diverse yet consistent across Europe. For example, unprofitability and lack of prospects were reported from Romania, Spain and Sweden. Both abandonment and intensification threatened the aesthetic value of the landscape, recreational opportunities and multiple values tied to traditional rural biotopes. People perceived intensification as decreasing human welfare through landscape homogenisation and loss of landscape quality.

The degree of visitor access to recreational areas is a dilemma, as visitor numbers should balance maintenance (Syrbe and Grunewald 2017). Restricting access due to the protection status of an area was one of the constraints to recreation in the analysed studies (see also Bell et al. 2007). Some studies reported that CES were no longer valued and acknowledged due to a loss of cultural identity. If designed and implemented carefully, protected areas can support traditions (Bell et al. 2007), and local development such as sustainable tourism may convince locals to continue their management practices (Kneafsey 2000; Mitchell and Buggey 2000). Agritourism is a common and successful tool for farm income diversification and improvement of small-scale farms, but also for rural development in general (Tew and Barbieri 2012; Bhatta and Ohe 2020). For tourists, it offers the benefits of education about agriculture and rural traditions alongside recreational opportunities (Tew and Barbieri 2012; Streifeneder and Dax 2020). Finally, traditional areas with extensive management on small farms support much more CES than highly modernised, homogeneous areas (Daniel et al. 2012).

Another important source of conflict was the perception of nature during recreational activities. When nature is not considered to have an intrinsic and distinct value but a decorative background for human activities, it may lead to environmental destruction (Nassauer 1995; Syrbe and Grunewald 2017). Perceptions may also differ between stakeholder groups about what is considered an aesthetic landscape or what recreational activities should be allowed in an area (Nowak-Olejnik et al. 2020; Winter et al. 2020). Structurally and functionally diverse areas have the potential to address such varying preferences, allowing more people to participate in recreational activities (Suárez et al. 2020). We reviewed several studies that compared the attitudes of residents to tourists or experts (e.g. Vinge and Flø 2015; Jacobsen and Tømmervik 2016; López-Rodríguez et al. 2019). Typically, locals preferred more open landscapes such as pastures and meadows. Natural afforestation was perceived as a threat to landscape aesthetics and recreation due to hindered views and reduced accessibility (see also Nassauer 1995). However, experts and foreign tourists considered forested areas closer to their natural state and an ‘ideal landscape’ with limited to no human impact (see also Schils et al. 2022).

Further major threats to CES were industrial activities and various forms of associated pollution. They can have a serious impact on human well-being and health (EEA 2015), as well as on ecosystems and their services (e.g. MEA 2005b; IPBES 2018). They further deteriorate natural capital (EEA 2015; IPBES 2018, 2019) and imperil the continuous provision of ecosystem services (EEA 2015), including CES.

Climate change threats were minor in our analysis, reported only six times as having adverse effects on recreation. The reduction of suitable areas for skiing and natural afforestation due to a rise in temperatures might hit the tourism industry substantially in the future (IPCC 2014). Mountainous regions might see a shift from mainly skiing tourism to summer or year-round tourism, although it is unknown whether such changes will be fully economically compensatory (IPCC 2014). It is unknown on what temporal and spatial scales these interactions between climate change and recreation will work (Monz et al. 2021).

### Threats from CES (recreation, tourism)

Ecosystems and the services they provide are created through the interplay of people with their environment (Villamagna et al. 2013; Comberti et al. 2015). If the balance between supply and demand is tipped, the ecosystem and associated ecosystem services become threatened (Villamagna et al. 2013; Maron et al. 2017). The earliest literature considered relevant to this systematic review were studies on the damaging effects of recreation and tourism on the ecosystem, which is how they are primarily discussed in conservation biology (Sumanapala & Wolf 2019). In our systematic review, the demand for recreation was by far the most important underlying cause for threats from CES.

We found touristic activities such as hiking, skiing and vehicle use (i.e. bikes, snowmobiles) to be the most important threats caused by recreation, mainly affecting vegetation, soil and wildlife. Typically, such effects become larger with increasing use, although the additional impact weakens at higher intensities (Cole 2004).

The threats to a region or landscape were typically related to the development of tourist facilities (e.g. roads and ski lifts) and the building of tourist accommodation. An increasing number of ‘day trips’ in rural and remote areas has increased the volume of transit traffic and the associated negative impact of roads and vehicles on the environment (EEA 2006). With tourism developing, farms disappear, and grassland management ceases, partly because work in the tourism industry is more lucrative and because tourist infrastructure is commonly built on agricultural fields and grasslands. Therefore, it is essential to monitor and minimise the impacts of tourism to develop the industry sustainably (EEA 2017). The overexploitation or destruction of ecosystems for purely monetary reasons must be prevented to guarantee the continued availability of ecosystem services (Syrbe and Grunewald 2017).

In the face of climate change and related increasingly snow-poor winters, the demand for artificial snow production will grow (Steiger et al. 2019). Alternatively, skiing facilities would need to be developed at higher altitudes (IPCC 2014), thus extending negative impacts into the highly sensitive high-Alpine zone (IPBES 2018). It will be essential to develop strategies for providing quality outdoor recreation in the face of the potential impacts caused by a changing climate (Ewert 1991; Fredman and Tyrväinen 2010; O’Toole et al. 2019). That will also include recognising locations where new types of activities are demanded (Ewert 1991; Askew and Bowker 2018).

### Negative feedback in CES

While recreation has clear positive links to mental and physical well-being (Hartig et al. 2014), it is not always beneficial for the ecosystem and may negatively affect the recreational and aesthetic quality of the area (Shin et al. 2010; Zhang et al. 2012; Syrbe and Grunewald 2017). We revealed studies on threats to or from CES during our systematic review, but few studies discussed them from both perspectives simultaneously. When agricultural activities cease, and the focus is shifted towards touristic uses, the capacity of the landscape to hold CES decreases. Increasing tourist infrastructure may make tourism more comfortable, but such structures reduce the cultural value of an area. Overcrowded places, high traffic flows or motorised recreation may clash with slower, more natural and more ‘relaxing’ tourism ideals in beautiful surroundings. For example, one of the reviewed studies (Getzner et al. 2016) suggested that an increased presence of tourist facilities is likely to reduce people’s length of stay in recreational areas. To reach a good balance (i.e. significant contributions from CES to biodiversity preservation and well-being) while avoiding the threats from increased pressure on the environment, an array of effective solutions, with careful design and construction of recreational areas, is needed (Huang and Confer 2009; Skokanová et al. 2016).

### Suggested solutions

The solutions for maintaining and improving CES delivery by permanent grasslands mainly concerned the prevention of land-use and land management changes caused by socio-economic factors. The suggested solutions comprised different institutional or socio-economic means that would tackle threats at all levels (i.e. also their underlying causes and consequences). Several papers suggested new agri-environmental programmes, which are especially important in marginal areas and should be incorporated into the new Common Agricultural Policy (CAP) (Carson et al. 2013). Such monetary support aims at supporting areas with limited productivity, ensuring continuous management of agricultural land that would otherwise be abandoned (MacDonald et al. 2000; Keenleyside and Tucker 2010; Molnár and Berkes 2018). Other possibilities include developing strategies to bring permanent residents back into rural areas and improving market access to counteract the loss of appreciation and abandonment due to unprofitability (Sedlacek et al. 2009; Baumann et al. 2011; Leal Filho et al. 2017). Agritourism has been suggested as a solution to maintain the connection between CES and traditional land use (see also Bell et al. 2007), connecting farming with tourism while simultaneously assuring the preservation of the landscape and farmers’ livelihoods during economic and social change (Keenleyside and Tucker 2010; Streifeneder and Dax 2020). Tourism is an important driver of economic development and an effective tool for biodiversity conservation (de Groot et al. 2005; EEA 2017). By adopting a more participatory approach, extensive traditional management practices could be maintained or restored (de Groot et al. 2005; Stenseke 2009; Conrad et al. 2011; Allan et al. 2015; IPBES 2018). This would include the maintenance of a multifunctional landscape to sustain its beauty and natural capital provision and to attract people for recreational activities (Raudsepp-Hearne et al. 2010; Beilin et al. 2014; IPBES 2018). An important example of such a multifunctional landscape in Europe is wood-pastures that can still be found across Europe (Plieninger et al. 2015b).

Improved communication and education was the second-most mentioned group of solutions and did not match any category in biodiversity literature. It encompassed a broad spectrum of knowledge transfer to tourists, the inclusion of relevant stakeholders in landscape planning activities and the education of residents on the value of grassland ecosystems and the services they provide. Combining scientific research and local knowledge about traditional management can ensure the continuity of management at appropriate intensities and can lead to renewed appreciation by the local population (Molnár et al. 2016). Especially in protected areas, detailed planning is needed to avoid mis- or under-management and protect the cultural landscape and its biodiversity (Stenseke 2009; Middleton 2013; Molnár and Berkes 2018). Furthermore, CES might be the most important communication channel to raise awareness for ecosystem protection due to its close links to human well-being (Comberti et al. 2015).

Regulations restricting industrial development can help maintain the landscape (Bürgi et al. 2005; see also Directive no. 2014/52/EU (EU 2014)). If designed carefully, they prevent the exploitation of sensitive areas for purely economic reasons and help ensure sustainable management. That is not to say that change should be prohibited altogether, as slow changes can prevent the loss of sense of place and identity (Bürgi et al. 2005). Recent progress in policy development suggests that economic growth at all costs is no longer the main priority (IPBES 2018). Increasing knowledge about environmental changes and their effects are helping to shape the institutional settings of the future (IPBES 2018).

Infrastructural solutions, such as improved access to rural regions (roads and paths), are a common tool in Europe to promote recreation in such areas (Bell et al. 2007). Somewhat counterintuitively, improving infrastructure for tourists to access remote areas can minimise the potential impacts of unrestrained recreation (Fredman and Tyrväinen 2010; Sumanapala and Wolf 2019). One reviewed paper suggested that improved accessibility can also enhance aesthetic experiences, despite the loss of untouched landscape through road or path construction (Schirpke et al. 2019). Landscape aesthetics were not limited to purely natural values such as a natural landscape or untouched snow, but also came from human-made values such as marked trails, look-out towers or ski lifts, as they allowed CES enjoyment in the first place (see also Soy Massoni et al. 2018).

It has been suggested by several studies, included in this systematic review and elsewhere (Cole and Monz 2002), that the limitation of visitor access would balance negative impacts of human trampling and disturbances. The temporal access limitation as a management option has been shown to be widely acceptable among visitors (Martin et al. 2009). However, trampling and related negative impacts cannot be avoided, and recovery is always slower than the impact time (Cole 2004). In fact, the biggest negative impacts are observed in newly disturbed rather than long disturbed areas (Cole 2004). With tools such as the Tourism Opportunity Spectrum (Butler and Waldbrook 2003), nature-based tourism may be planned and managed more strategically to favour CES and biodiversity simultaneously. With so-called “honey-pots” (Pescott and Stewart 2014, p. 14) visitors are lured away from vulnerable areas. The provision of alternative recreational activities nearby could further be combined with the restoration of trails as a fast and effective measure to minimise impact (Eagan et al. 2000). The long-term effects of restoration have also been shown to successfully mitigate the negative impacts of ski piste construction (Hudek et al. 2020), even if not to natural levels (Caprio et al. 2016). Ideally, negative impacts are anticipated and addressed during the planning phase of new recreational areas and tailored to the specific circumstances of an area (Butler and Waldbrook 2003; Huang and Confer 2009).

### Limitations, research gaps and recommendations

In the reviewed papers, the study sites and locations were rarely described in-depth, which hindered the extraction of relevant information (e.g. grassland type and spatial scale). Although highly relevant for CES, the exact spatial scale was rarely mentioned or identifiable implicitly (Tengberg et al. 2012; Scholte et al. 2015). Methodological differences in the assessment and categorisation of threats made it challenging to classify and compare studies (see also Milcu et al. 2013) and thus to draw conclusions on their prevalence in European permanent grasslands. Underlying causes and direct threats were not always easy to identify, and were based on consensus and thematic considerations rather than exact definitions. Indirect drivers are often hierarchical and could be traced back to multiple levels, and thus could only be considered in general and overarching terms (Contreras-Hermosilla 2000; Bürgi et al. 2005). Furthermore, threats to CES were often mentioned as negative impacts to the ecosystem rather than to the service itself. Future research on threats should therefore be designed and interpreted targeting the impact on the service rather than on the ecosystem.

Despite a recent increase in CES research there are still considerable research gaps regarding threats surrounding CES, especially in countries with high rates of agricultural intensification (Carson et al. 2013; see also Supplementary Fig. S3) and urban and other commercial development (Perpiña Castillo et al. 2019). A lack of application of a widely used framework in the individual studies made the comparison or integration of cases difficult, and hampered the making of general conclusions. The cascade model of ecosystem services (Haines-Young and Potschin 2018) or the IPBES global assessment (IPBES 2019) are examples of widely used approaches and should be considered when addressing threats to CES in future studies.

In this systematic review, we found three major aspects not present in the classification systems we used for comparison (see Data analysis). “Social attitudes” in the direct threats, and “communication” and “restoration” in the suggested solutions were views not previously categorised in literature. One plausible reason could be that the classification systems used targeted biodiversity, while this systematic review addressed CES. We recommend developing categories that specifically take cultural and social aspects (i.e. attitudes, perceptions) into account. We consider CES to be the crucial link in balancing supply and demand of ecosystem services, not least because the foundation of values, attitudes, and beliefs lies in culture and traditions and directly affects how ecosystems and their services are perceived (Nassauer 1995; Contreras-Hermosilla 2000).

Existing habitat categorisations do not focus on ecological processes and services, including landscape aesthetics. Therefore, it is necessary to explore the delineation of habitat qualities for CES, and to improve the understanding of how ecological processes provide continued CES supply (Allan et al. 2015). Carefully planned and well-managed tourism might prevent the need for the restriction of recreational activities that in ‘normal’ circumstances can harm ecological processes (Butler and Waldbrook 2003).

## Conclusion

We identified and analysed the studies relevant for this systematic review and found that conserving and improving CES would need a multi-actor approach in which rural development and traditional knowledge are integrated into permanent grassland management. We also identified knowledge gaps that need to be addressed in future research.

Further research evidence would improve our understanding of the role of CES within the context of new challenges. The COVID-19 pandemic, which led to the restriction of many leisure activities such as cultural or sports events, has been shown to shift the demand for recreation towards outdoor activities (Day 2020; Venter et al. 2020). Nature can serve as a refuge, reducing stress and increasing human well-being, especially when life is otherwise restricted and limited to urban areas (de Groot et al. 2005; Hartig et al. 2014; Samuelsson et al. 2020). Yet, this increasing demand for recreation in natural and especially remote areas will increase the pressure on the ecosystem and its services. That is not a new trend (EEA 2017) but may be reinforced by the recent situation. It is, therefore, essential to find strategies to minimise impacts caused by recreation through continuous, interdisciplinary scientific research (Sumanapala and Wolf 2019) and in close dialogue with relevant stakeholders (Chan et al. 2012; Bennett et al. 2015; Díaz et al. 2015).

Climate change is another global challenge that has effects on CES. We found surprisingly few studies discussing the threats posed by climate change, and most concerned winter recreation. However, the increasing frequency of extreme events such as droughts and associated threats will also have devastating effects on habitats and their ecosystem services (IPBES 2018). According to IPBES (2018, p. 392), climate change “is likely to become one of the most important drivers in the future, especially in combination with other drivers”. However, a changing climate may also bring new recreation opportunities, as formerly inhospitable areas become more accessible (IPCC 2014; Askew and Bowker 2018; Monz et al. 2021). To avoid negative effects and on ecosystem services conflicts between different recreation types, careful planning of recreational areas will be needed (Winter et al. 2020).

We call for the consequent incorporation of CES into all future ecosystem service frameworks and policies (see also Daniel et al. 2012; Díaz et al. 2018). CES can be a powerful approach to support the aim of the new EU biodiversity strategy to protect approximately one-third of Europe and to restore nature (EC 2020). Integrating CES into policy frameworks offers opportunities for improved understanding and assessment of how ecosystems can benefit human well-being (Daniel et al. 2012; Hirons et al. 2016; Kosanic and Petzold 2020). Culture plays a crucial role in (re-)connecting people with nature (Hirons et al. 2016; Díaz et al. 2018) and is related to the well-being of people (Gobster et al. 2007; Hernández-Morcillo et al. 2013; Hirons et al. 2016). It might further divert the attention from materialistically focused policies and value systems to more socially and spiritually oriented approaches (Hirons et al. 2016). With the proper tools in place to mitigate (future) negative impacts, the continued provision of CES can be guaranteed.

## Supplementary Information

Below is the link to the electronic supplementary material.

Supplementary file1 (PDF 2698 KB)
