# Supplementary material for: Cultural ecosystem services in European grasslands: A systematic review of threats
Source: Ambio. 2022 Jul 6;51(12):2462–77. doi: 10.1007/s13280-022-01755-7 (PMC9257557; doi:10.1007/s13280-022-01755-7)
Supplement: Supplementary file 1 — Supplementary file1 (PDF 2698 KB) [file 13280_2022_1755_MOESM1_ESM.pdf]

Ambio

Supplementary Information
This supplementary information has not been peer reviewed.

Title: **Cultural ecosystem services in European grasslands: a systematic review of threats**

Supplementary material - Appendix S1

Supplementary material for literature search, selection and extraction.

Table S1. Search strings used in the literature search within Scopus and CAB abstract databases for grassland studies (search performed on 5 November 2019).

Grass	dryland? or grass* or grazed or grazing or hayfield? or hay field? or haymeadow? or herbaceous or herbage? or meadow? or pasture? or rangeland? or range land? or ryegrass* or silvopast* or steppe or steppes or sward? or woodpast*
Europe	exp europe/ or (albania or andorra or austria or belarus or belgium or bosnia or british isles or bulgaria or croatia or cyprus or czech republic or czechia or czechoslovakia or denmark or england or estonia or estonian soviet socialist republic or estonian ssr or europe* or finland or flanders or france or german democratic republic or german federal republic or germany or great britain or greece or hercegovina or herzegovina or hungary or iceland or ireland or irish republic or italy or kosovo or latvia or latvian ssr or liechtenstein or lithuania or lithuanian soviet socialist republic or lithuanian ssr or luxemb?urg or macedonia or moldova or monaco or montenegro or netherlands or norway or poland or portugal or romania or san marino or scotland or serbia or slovakia or slovenia or spain or sweden or switzerland or uk or ukraine or united kingdom or wales or wallonia or walloon or yugoslavia) not (new england or new south wales)
Recreation	ecotouris* or leisur* or recreati* or touris* or outdoor? or cultural or bicycle? or bike? or biking or birding or birdwatch* or (bird? adj1 watch*) or climb* or cyclist? or geocach* or hiker? or hiking or hunting or nature trail? or relax* or riding or rider? or travel* or trekker? or trekking or walk* or sport* or physical activit* or social activit*
Landscape aesthetic	aesthetic* or esthetic* or beauty or landscape? or photo? or photograph? or photographer? or scenery or sceneries or visitor? or social media or facebook or twitter or instagram or snapchat or pinterest or tumblr or whatsapp or youtube

Paper selection and data extraction

As a first step, titles and abstracts were screened on relevance and filtered based on a set of exclusion criteria. In the first round of screening, 13 523 papers were excluded (see Fig. S1). The exclusion criteria included:

- not in the English language,
- outside the biogeographic zones of interest: Alpine, Atlantic, Boreal, Continental, Mediterranean or Pannonian,
- outside the following countries in Europe: Member states of the EU-28 or Albania, Belarus, Bosnia Herzegovina, Kosovo, Macedonia, Moldova, Montenegro, Norway, Serbia, Switzerland or Ukraine,
- unit of study is not permanent grassland,
- the outcome is not on the cultural ecosystem services of interest (recreation or landscape aesthetics),
- reviews,
- modelling studies, and
- experiments under controlled conditions.

As a second step, the full texts of the remaining 196 papers were scanned, of which 71 papers were selected that contained at least one of the following aspects:

- threats to CES and permanent grasslands,
- threats to grasslands from CES,
- solutions to prevent or reverse threats.

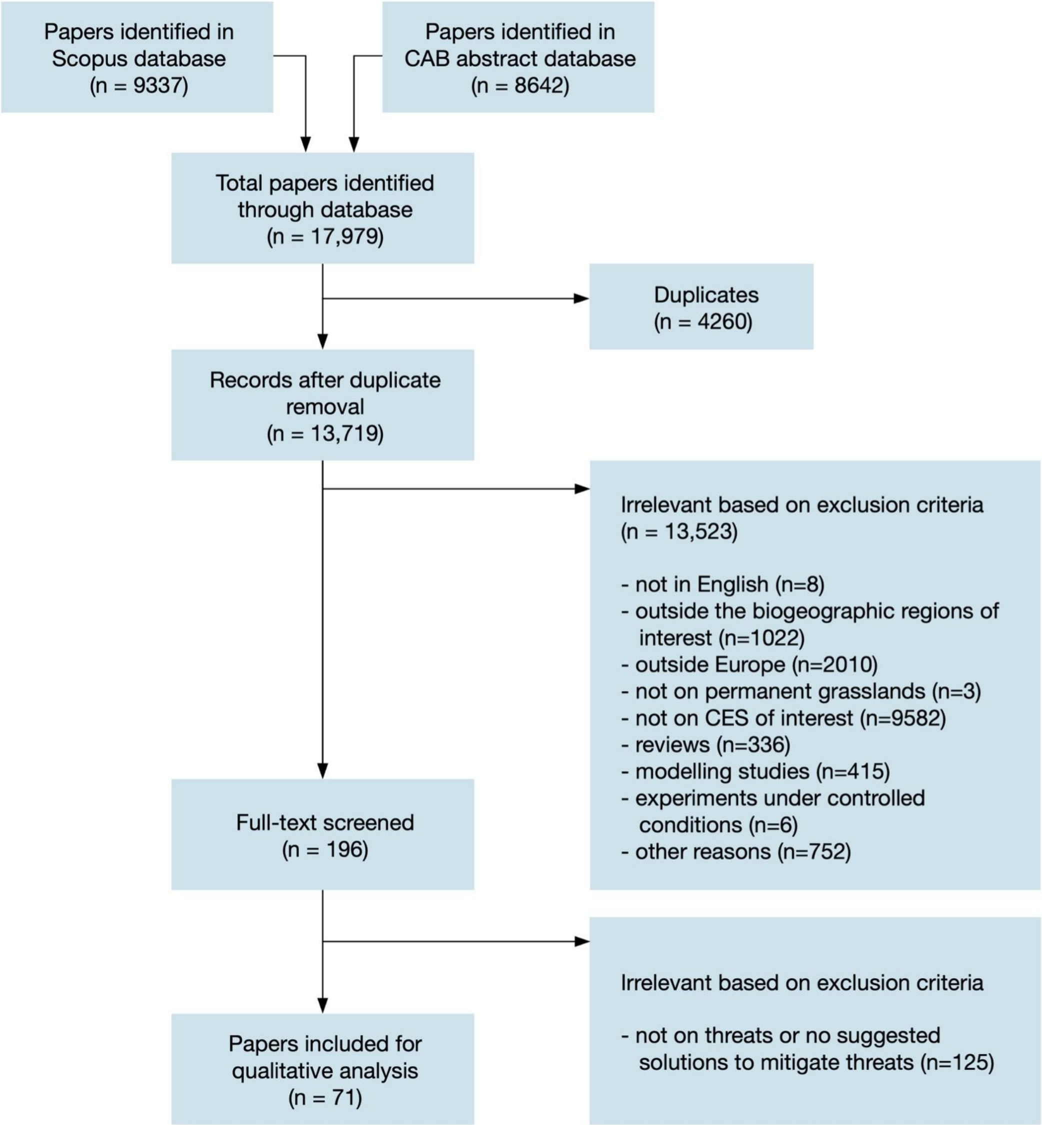

Figure S1. Flowchart of the data extraction.

Table S2a. Data extraction form for general characteristics.

Attribute	Values, description
Nr	Unique number for each study
Title	Full title of the study
Authors	Authors of the study
Year	Year of publication
DOI	DOI of the publication
Country	Country where the study was conducted
Region of the study	Name of the region or place
Spatial scale	Country
	Regional
	Landscape
	Plot
Biogeographical region	Alpine
	Arctic
	Atlantic
	Black Sea
	Boreal
	Continental
	Mediterranean
	Pannonian
Grassland: Details	Type of grassland under study as a quote from the text
Grassland type: Broad category	Heathland
	Improved (reseeded, fertilised)
	Meadow
	Moss-lichen vegetation
	Natural grasslands
	Orchard
	Pasture
	Peatland
	Rangeland
	Simplified grassland type
	Wood-pasture
Study approach	Field study
	Stakeholder survey
	Regional study
	Other (specified)
Suitable for review	Yes/No

Table S2b. Data extraction form for the threats analysis.

**Attribute**	**Description**
Author	Authors of the study
Study region	Specific region the study was conducted in case of multiple, independent studies
Underlying causes: Details	Details from the paper, preferably quotes; separate for threats TO and threats FROM CES
Underlying causes: Broad category	Category defined by IPBES (if an equivalent existed); separate for threats TO and threats FROM CES
Direct threats: Details	Details from the paper, preferably quotes; separate for threats TO and threats FROM CES
Direct threats: Broad category	Category defined by IUCN-CMP (if an equivalent existed); separate for threats TO and threats FROM CES
Consequences to CES: Details	Details from the paper, preferably quotes; separate for threats TO and threats FROM CES
Consequences to CES: Broad category	Category, developed post-hoc; separate for threats TO and threats FROM CES
Suggested solutions: Details	Details from the paper, preferably quotes for any given threat level (underlying cause, direct threat, consequences); separate for threats TO and threats FROM CES
Suggested solutions: Broad category	Category, developed post-hoc; separate for threats TO and threats FROM CES

Table S3. Comparison of classification systems in this systematic review and in IPBES (IPBES 2018) and IUCN-CMP (IUCN-CMP 2019). Effects show whether a factor was overabundant (+), lacking (-) or mixed (+/-) in our systematic review.

<table>
<tr><th colspan="3">Underlying causes</th></tr>
<tr><th>This systematic review</th><th>IPBES</th><th>Effects</th></tr>
<tr><td>institutional</td><td>institutional</td><td>+/-</td></tr>
<tr><td>demographic</td><td>demographic</td><td>-</td></tr>
<tr><td>no equivalent</td><td>scientific & technological</td><td></td></tr>
<tr><td>economic</td><td rowspan="3">economic</td><td>+</td></tr>
<tr><td>socio-economic</td><td>+/-</td></tr>
<tr><td>infrastructural</td><td>+/-</td></tr>
<tr><td>cultural</td><td>cultural & religious</td><td>+/-</td></tr>
<tr><td>climate change</td><td>no equivalent</td><td>+/-</td></tr>
</table>

Table S3. *continued*

Direct threats		
**This systematic review**	**IUCN-CMP**	**Effects**
land-use change: building up	residential & commercial development	+
development of tourist facilities		+
building of tourist accommodations		+
management change: intensification	agriculture & aquaculture	+
land-use change: afforestation		+
industrial/economic activities: mining & quarrying	energy production & mining	+
industrial/economic activities: wind farms & solar panels		+
land-use change: linear infrastructure	transportation & service corridors	+/-
(social attitude: poor recreational services)		-
hunting, gathering plants	biological resource use	+
tourism	human intrusions & disturbance	+/-
land-use change: abandonment	natural system modifications	-
no equivalent	invasive & problematic species	
Management change: grass burning	pollution	+
industrial/economic activities: air pollution		+
industrial/economic activities: military exercise		+
industrial/economic activities: waste deposition		+
no equivalent	geological events	
(natural threats: decreased glaciers & snow)	climate change	+
(natural threats: spontaneous afforestation)		+
natural threats: natural hazards	*no equivalent*	+
social attitude	*no equivalent*	-

Table S3. *continued*

Consequences	
**This systematic review**	**Threat direction**
Loss of landscape aesthetics	Threats to CES
Loss of recreational appeal	
Restricted recreational activities	
Hampered tourism development	
Land degradation	Threats from CES
Biodiversity loss	
Loss of traditional cultural landscape	
Negative impact of tourism	
Negative impact on aesthetics	
Negative impact on locals	

Suggested solutions		
**This systematic review**	**IPBES**	**Threat direction**
institutional	institutional	Threats to/from CES
socio-economic	economic	Threats to CES
infrastructural		
communication	*no equivalent*	Threats to CES
restoration	*no equivalent*	Threats to/from CES

References

IPBES (Intergovernmental Science-Policy Platform on Biodiversity and Ecosystem Services). 2018. *The IPBES regional assessment report on biodiversity and ecosystem services for Europe and Central Asia*. eds. M. Rounsevell, M. Fischer, A. Torre-Marín Rando, and A. Mader. Bonn: Secretariat of the Intergovernmental Science-Policy Platform on Biodiversity and Ecosystem Services.

IUCN-Conservation Measures Partnership (IUCN-CMP). 2019. IUCN-CMP unified classification of direct threats v3.2. Retrieved 13 November, 2020, from https://www.iucnredlist.org/resources/threat-classification-scheme.

Supplementary material - Appendix S2

Full list of included publications.

Aall, C., and K.G. Høyer. 2005. Tourism and climate change adaptation-the Norwegian case. In *Tourism, Recreation and Climate Change*, eds. C.M. Hall and J. Higham, 209–223. London: Channelview Press.

Abrudan, I., and D. Turnock. 1999. A rural development strategy for the Apuseni Mountains, Romania. *GeoJournal* 46: 319–336.

Bastian, O., C. Stein, G. Lupp, J. Behrens, C. Renner, and K. Grunewald. 2015. The appreciation of nature and landscape by tourism service providers and visitors in the Ore Mountains (Germany). *Landscape Online* 41: 1–23. doi:10.3097/LO.201541.

Bastian, O., R.U. Syrbe, J. Slavik, J. Moravec, J. Louda, B. Kochan, N. Kochan, S. Stutzriemer, et al. 2017. Ecosystem services of characteristic biotope types in the Ore Mountains (Germany/Czech Republic). *International Journal of Biodiversity Science, Ecosystem Services and Management* 13: 51–71. doi:10.1080/21513732.2016.1248865.

Bernués, A., T. Rodríguez-Ortega, R. Ripoll-Bosch, and F. Alfnes. 2014. Socio-cultural and economic valuation of ecosystem services provided by Mediterranean mountain agroecosystems. *PLoS ONE* 9, e102479. doi:10.1371/journal.pone.0102479.

Bernués, A., E. Tello-García, T. Rodríguez-Ortega, R. Ripoll-Bosch, and I. Casasús. 2016. Agricultural practices, ecosystem services and sustainability in High Nature Value farmland: Unraveling the perceptions of farmers and nonfarmers. *Land Use Policy* 59: 130–142. doi:10.1016/j.landusepol.2016.08.033.

Brambilla, M., P. Pedrini, A. Rolando, and D.E. Chamberlain. 2016. Climate change will increase the potential conflict between skiing and high-elevation bird species in the Alps. *Journal of Biogeography* 43: 2299–2309. doi:10.1111/jbi.12796.

Buza, M., L. Dimen, G. Pop, and D. Turnock. 2001. Environmental protection in the Apuseni Mountains: The role of Environmental Non-Governmental Organisations (ENGOs). *GeoJournal* 54: 631–653.

Caprio, E., D.E. Chamberlain, M. Isaia, and A. Rolando. 2011. Landscape changes caused by high altitude ski-pistes affect bird species richness and distribution in the Alps. *Biological Conservation* 144: 2958–2967. doi:10.1016/j.biocon.2011.08.021.

Conradi, T., K. Strobl, A.L. Wurfer, and J. Kollmann. 2015. Impacts of visitor trampling on the taxonomic and functional community structure of calcareous grassland. *Applied Vegetation Science* 18: 359–367. doi:10.1111/avsc.12164.

Daugstad, K., M.F. Mier, and L. Peña-Chocarro. 2014. Landscapes of transhumance in Norway and Spain: Farmers' practices, perceptions, and value orientations. *Norsk Geografisk Tidsskrift* 68: 248–258. doi:10.1080/00291951.2014.927395.

Dobay, G., B. Dobay, S.E. Falusi, S. Hajnáczki, K. Penksza, Z. Bajor, R. Lampert, G. Bakó, et al. 2017. Effects of sport tourism on temperate grassland communities (duna-ipoly national park, Hungary). *Applied Ecology and Environmental Research* 15: 457–472. doi:10.15666/aeer/1501_457472.

Faccioni, G., E. Sturaro, M. Ramanzin, and A. Bernués. 2019. Socio-economic valuation of abandonment and intensification of Alpine agroecosystems and associated ecosystem services. *Land Use Policy* 81: 453–462. doi:10.1016/j.landusepol.2018.10.044.

Filepné Kovács, K., G.G. Nagy, and L. Kollányi. 2012. Evaluation of rural landscape functions based on domestic case study. *Applied Ecology and Environmental Research* 10: 17–30.

Fish, R., A. Church, C. Willis, M. Winter, J.A. Tratalos, R. Haines-Young, and M. Potschin. 2016. Making space for cultural ecosystem services: Insights from a study of the UK nature improvement initiative. *Ecosystem Services* 21: 329–343. doi:10.1016/j.ecoser.2016.09.017.

Früh-Müller, A., S. Hotes, L. Breuer, V. Wolters, and T. Koellner. 2016. Regional patterns of ecosystem services in cultural landscapes. *Land* 5, 17. doi:10.3390/land5020017.

Gallet, S., and F. Rozé. 2001. Resistance of Atlantic Heathlands to trampling in Brittany (France): influence of vegetation type, season and weather conditions. *Biological Conservation* 97: 189–198.

Gallet, S., and F. Rozé. 2002. Long-term effects of trampling on Atlantic Heathland in Brittany (France): resilience and tolerance in relation to season and meteorological conditions. *Biological Conservation* 103: 267–275.

Gąsienica Byrcyn, W. 1992. The history and present role of the Tatra National Park. *Mountain Research & Development* 12: 205–210. doi:10.2307/3673791.

Getzner, M., B. Färber, and C. Yamu. 2016. 2D versus 3D: The relevance of the mode of presentation for the economic valuation of an alpine landscape. *Sustainability* 8, 591. doi:10.3390/su8060591.

Gómez-Limón, F.J., and J.V. de Lucio. 1995. Recreational activities and loss of diversity in grasslands in Alta Manzanares Natural Park, Spain. *Biological Conservation* 74: 99–105. doi:10.1016/0006-3207(95)00018-Y.

Górski, P. 2009. The effects of hikers' paths on the distribution of liverworts in the Tatra Mountains (Western Carpathians). *Cryptogamie, Bryologie* 30: 229–242.

Grabherr, G. 1982. The impact of trampling by tourists on a high altitudinal grassland in the Tyrolean Alps, Austria. *Vegetatio* 48: 209–217. doi:10.1007/BF00055262.

Hasund, K.P., M. Kataria, and C.J. Lagerkvist. 2011. Valuing public goods of the agricultural landscape: A choice experiment using reference points to capture observable heterogeneity. *Journal of Environmental Planning and Management* 54: 31–53. doi:10.1080/09640568.2010.502753.

Hausner, V.H., G. Brown, and E. Lægreid. 2014. Effects of land tenure and protected areas on ecosystem services and land use preferences in Norway. *Land Use Policy* 49: 446–461. doi:10.1016/j.landusepol.2015.08.018.

Honrado, J.P., A. Lomba, P. Alves, C. Aguiar, T. Monteiro-Henriques, Y. Cerqueira, P. Monteiro, and F. Barreto Caldas. 2017. Conservation Management of EU Priority Habitats after Collapse of Traditional Pastoralism: Navigating Socioecological Transitions in Mountain Rangeland. *Rural Sociology* 82: 101–128. doi:10.1111/ruso.12111.

Jacobsen, J.K.S., and H. Tømmervik. 2016. Leisure traveller perceptions of iconic coastal and fjord countryside areas: Lush naturalness or remembrance of agricultural times past? *Land Use Policy* 54: 38–46. doi:10.1016/j.landusepol.2016.01.013.

Kammer, P.M. 2002. Nature Conservation Floristic changes in subalpine grasslands after 22 years of artificial snowing. *Journal of Nature Conservation* 10: 109–123.

Kňazovičová, L., S. Chasníková, J. Novák, and P. Barančok. 2018. Impacts of ski pistes preparation and ski tourism on vegetation. *Ekológia Bratislava* 37: 152–163. doi:10.2478/eko-2018-0014.

Knežević, R., R. Grbac Žiković, and M. Magdić. 2011. Factors of sustainable tourism development in the Ogulin-Plaški Micro Region. In *Sustainable Tourism: Socio-Cultural, Environmental and Economics Impact*, ed. J. Perić, 187–203. University of Rijeka, Croatia: Faculty of Tourism and Hospitality Management

Kycko, M., B. Zagajewski, S. Lavender, E. Romanowska, and M. Zwijacz-Kozica. 2018. The impact of tourist traffic on the condition and cell structures of alpine swards. *Remote Sensing* 10, 220. doi:10.3390/rs10020220.

Kyriazopoulos, A.P., G. Arabatzis, E.M. Abraham, Z.M. Parissi, G. Kyprianou, and K. Soutsas. 2015. Citizens perceptions of current land management in the rangelands of the Akama Peninsula, Cyprus. A first approach. *Journal of Environmental Protection and Ecology* 16: 1565–1572.

Kyriazopoulos, A.P., G. Arabatzis, E.M. Abraham, and Z.M. Parissi. 2013. Threats to Mediterranean rangelands: A case study based on the views of citizens in the Viotia prefecture, Greece. *Journal of Environmental Management* 129: 615–620. doi:10.1016/j.jenvman.2013.08.035.

Laiolo, P., and A. Rolando. 2005. Forest bird diversity and ski-runs: A case of negative edge effect. *Animal Conservation* 7: 9–16. doi:10.1017/S1367943004001611.

Lamarque, P., P. Meyfroidt, B. Nettier, and S. Lavorel. 2014. How ecosystem services knowledge and values influence farmers' decision-making. *PLoS ONE* 9, e107572. doi:10.1371/journal.pone.0107572.

Lamarque, P., U. Tappeiner, C. Turner, M. Steinbacher, R. D. Bardgett, U. Szukics, M. Schermer, and S. Lavorel. 2011. Stakeholder perceptions of grassland ecosystem services in relation to knowledge on soil fertility and biodiversity. *Regional Environmental Change* 11: 791–804. doi:10.1007/s10113-011-0214-0.

Lee, J.J. 2019. A walk in the long grass: agriculture, aesthetics, and wildness on Hampstead Heath. *Landscape Research* 44: 846–856. doi:10.1080/01426397.2018.1427708.

Lee, J. 2007. Experiencing landscape: Orkney hill land and farming. *Journal of Rural Studies* 23: 88–100. doi:10.1016/j.jrurstud.2006.05.002.

Lindemann-Matthies, P., R. Briegel, B. Schüpbach, and X. Junge. 2010. Aesthetic preference for a Swiss alpine landscape: The impact of different agricultural land-use with different biodiversity. *Landscape and Urban Planning* 98: 99–109. doi:10.1016/j.landurbplan.2010.07.015.

López-Rodríguez, A., R. Escribano-Bombín, V. Hernández-Jiménez, and S. Bell. 2019. Perceptions of ecological and aesthetic quality by natural resource professionals and local people. A qualitative exploration in a mountainous landscape (La Rioja, Spain). *Landscape Research* 44: 241–255. doi:10.1080/01426397.2018.1446073.

Marín-Yaseli, M.L., and T. Lasanta Martínez. 2003. Competing for meadows. A case study on tourism and livestock farming in the Spanish Pyrenees. *Mountain Research and Development* 23: 169–176.

Müller, S.M., J. Peisker, C. Bieling, K. Linnemann, K. Reidl, and K. Schmieder. 2019. The importance of cultural ecosystem services and biodiversity for landscape visitors in the biosphere reserve Swabian Alb (Germany). *Sustainability* 11, 2650. doi:10.3390/su11092650.

Olsson, E.G.A., S.K. Hanssen, and K. Rønningen. 2004. Different conservation values of biological diversity? A case study from the Jotunheimen mountain range, Norway. *Norsk Geografisk Tidsskrift* 58: 204–212. doi:10.1080/00291950410002700.

Paderewski, J., and D. Sienkiewicz-Paderewska. 2010. Multidimensional scaling for describing the risks for grasslands ecosystems located in the Polish Landscape Parks. In *Grassland in a changing world*, eds. H.

Schnyder, J. Isselstein, F. Taube, K. Auerswald, J. Schellberg, M. Wachendorf, A. Herrmann, M. Gierus et al., 723–725. Zürich: European Grassland Federation.

Pătru-Stupariu, I., C.A. Tudor, M.S. Stupariu, A. Buttler, and A. Peringer. 2016. Landscape persistence and stakeholder perspectives: The case of Romania's Carpathians. *Applied Geography* 69: 87–98. doi:10.1016/j.apgeog.2015.07.015.

Patthey, P., S. Wirthner, N. Signorell, and R. Arlettaz. 2008. Impact of outdoor winter sports on the abundance of a key indicator species of alpine ecosystems. *Journal of Applied Ecology* 45: 1704–1711. doi:10.1111/j.1365-2664.2008.01547.x.

Pleșoianu, D., and V. Simionescu. 2016. Tourism, economic activity with great potential but still insufficient valued in settlements of the Lagoon Complex Razim-Sinoe. *Scientific Papers Series Management, Economic Engineering in Agriculture and Rural Development* 16: 393–404.

Quintas-Soriano, C., A.J. Castro, H. Castro, and M. García-Llorente. 2016. Impacts of land use change on ecosystem services and implications for human well-being in Spanish drylands. *Land Use Policy* 54: 534–548. doi:10.1016/j.landusepol.2016.03.011.

Raatikainen, K.J., and E.S. Barron. 2017. Current agri-environmental policies dismiss varied perceptions and discourses on management of traditional rural biotopes. *Land Use Policy* 69: 564–576. doi:10.1016/j.landusepol.2017.10.004.

Rewitzer, S., R. Huber, A. Grêt-Regamey, and J. Barkmann. 2017. Economic valuation of cultural ecosystem service changes to a landscape in the Swiss Alps. *Ecosystem Services* 26: 197–208. doi:10.1016/j.ecoser.2017.06.014.

Runnström, M. C., R. Ólafsdóttir, J. Blanke, and B. Berlin. 2019. Image analysis to monitor experimental trampling and vegetation recovery in Icelandic plant communities. *Environments* 6, 99. doi:10.3390/environments6090099.

Săftoiu, L.C., and A.V. Munteanu. 2014. Tourist Activities-Disturbance Factors of Natural Environment in Ciucaș Massif. *Journal of Environmental and Tourism Analyses* 2: 97–114.

Schaminée, J.H.J., and M.H. Meertens. 1992. The influence of human activities on the vegetation of the subalpine zone of the Monts du Forez (Massif Central, France). *Preslia* 64: 327–342.

Schermer, M., I. Darnhofer, K. Daugstad, M. Gabillet, S. Lavorel, and M. Steinbacher. 2016. Institutional impacts on the resilience of mountain grasslands: An analysis based on three European case studies. *Land Use Policy* 52: 382–391. doi:10.1016/j.landusepol.2015.12.009.

Schirpke, U., A. Altzinger, G. Leitinger, and E. Tasser. 2019. Change from agricultural to touristic use: Effects on the aesthetic value of landscapes over the last 150 years. *Landscape and Urban Planning* 187: 23–35. doi:10.1016/j.landurbplan.2019.03.004.

Schüpbach, B., A. Grünig, and T. Walter. 2004. Grassland and landscape aesthetics. In *Land use systems in grassland dominated regions*, eds. A. Lüscher, B. Jeangros, O. Huguenin, M. Lobsiger, N. Millar, and D. Suter, 186–188. Reading: European Grassland Federation.

Soliva, R., J. Bolliger, and M. Hunziker. 2010. Differences in preferences towards potential future landscapes in the Swiss Alps. *Landscape Research* 35: 671–696. doi:10.1080/01426397.2010.519436.

Soy Massoni, E.S., D. Varga, M. Sez, and J. Pintt. 2016. Exploring aesthetic preferences in rural landscapes and the relationship with spatial pattern indices. *Journal of Landscape Ecology* 9: 5–21.

Staglianò, N., G. Argenti, A. Albertosi, E. Bianchetto, and A. Pardini. 2005. Mediterranean pastures management by local cattle breeds for the valorization of typical products and for the development of nature tourism. In *Options Méditerranéennes, Series A*, 239–244.

Stanciu, M., R. Blaj, and M. Dumitru. 2014. Promoting Natura 2000 network benefits for local communities by practicing ecotourism and agrotourism. *Scientific Papers Series Management, Economic Engineering in Agriculture and Rural Development* 14: 349–356.

Steinbauer, M. J., J. Kreyling, C. Stöhr, and V. Audorff. 2018. Positive sport–biosphere interactions? — Cross-country skiing delays spring phenology of meadow vegetation. *Basic and Applied Ecology* 27: 30–40. doi:10.1016/j.baae.2017.10.003.

Stenseke, M. 2006. Biodiversity and the local context: linking seminatural grasslands and their future use to social aspects. *Environmental Science and Policy* 9: 350–359. doi:10.1016/j.envsci.2006.01.007.

Stypiński, P., and D. Sienkiewicz-Paderewska. 2007. The protection and conservation of grasslands in the Polish Landscape Parks. In *Permanent and temporary grassland. Plant, environment and economy*, eds. A. De Vliegher and L. Carlier, 520–523. Zürich: European Grassland Federation.

Tardieu, L., S. Roussel, J.D. Thompson, D. Labarraque, and J.M. Salles. 2015. Combining direct and indirect impacts to assess ecosystem service loss due to infrastructure construction. *Journal of Environmental Management* 152: 145–157. doi:10.1016/j.jenvman.2015.01.034.

Tempesta, T., and D. Vecchiato. 2018. The value of a properly maintained hiking trail network and a traditional landscape for mountain recreation in the dolomites. *Resources* 7, 86. doi:10.3390/resources7040086.

Tokarczyk, N. 2018. Challenges for the conservation of semi-natural grasslands in mountainous national parks - Case studies from the Polish Carpathians. *Carpathian Journal of Earth and Environmental Sciences* 13: 187–198. doi:10.26471/cjees/2018/013/017.

Tomczyk, A.M., and M.W. Ewertowski. 2016. Recreational trails in the Poprad Landscape Park, Poland: the spatial pattern of trail impacts and use-related, environmental, and managerial factors. *Journal of Maps* 12: 1227–1235. doi:10.1080/17445647.2015.1088751.

Torralba, M., N. Fagerholm, T. Hartel, G. Moreno, and T. Plieninger. 2018. A social-ecological analysis of ecosystem services supply and trade-offs in European wood-pastures. *Science Advances* 4, eaar2176. doi:10.1126/sciadv.aar2176.

Vinge, H., and B.E. Flø. 2015. Landscapes Lost? Tourist Understandings of Changing Norwegian Rural Landscapes. *Scandinavian Journal of Hospitality and Tourism* 15: 29–47. doi:10.1080/15022250.2015.1010283.

Vojáček, O., and J. Louda. 2017. Economic value of ecosystem services in the Eastern Ore Mountains. *Ekonomie* 20: 4–18. doi:10.15240/tul/001/2017-3-001.

Wytrzens, H.K., and C. Mayer. 1999. Multiple use of alpine grassland in Austria and the implications for agricultural policy. *Bodenkultur* 50: 251–261.

Supplementary material - Appendix S3

Additional results.

Outline of the evidence

Seventy-one of the 77 analysed studies were published after 2000, with a considerable increase in the last six years (Fig. S2). The earliest relevant study was published in 1982 (Grabherr 1982), investigating the effects of trampling on a distance gradient from hiking paths. The subsequent papers in 1992 and 1995 (Gąsienica Byrcyn 1992; Schaminée and Meertens 1992; Gómez-Limón and de Lucio 1995) elaborated similar study questions. The negative impacts of human activities on the aesthetic quality of a landscape was first mentioned in a paper published in 1999 (Wytrzens and Mayer 1999), with an increasing number of studies on this topic in the past decade.

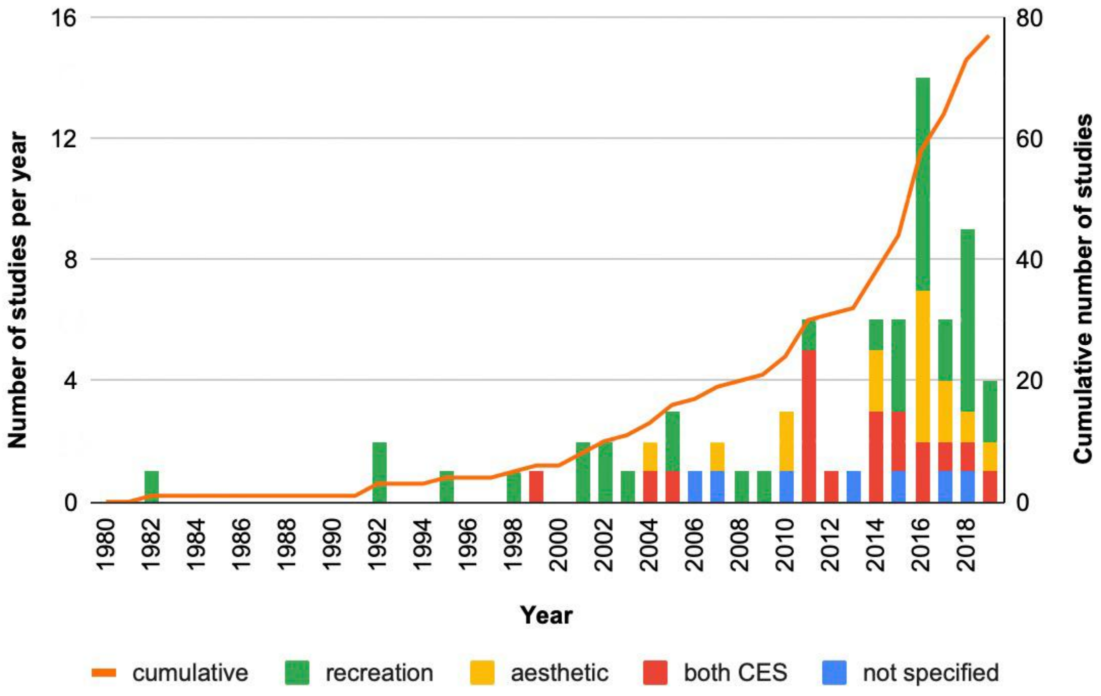

Figure S2. Number of relevant studies since 1980 on threats related to cultural ecosystem services (CES) in permanent grassland.

The included studies stem from 20 European countries (Fig. S3; a complete list of paper characteristics can be found in Table S4), with most study locations situated in Spain (n=10; note that one paper can contain more than one study location), Poland, Romania, Norway, Italy and France (each n=7). No studies were conducted in Ireland, the Benelux countries, Ukraine or northeastern Europe. There was no correlation between the number of study sites and the percentage cover of permanent grassland on the utilised agricultural area (Fig. S3) or management intensity (Fig. S4, where the amount of manufactured fertiliser applied is used as a proxy for management intensity).

The most studied grassland types were natural grasslands (n=30; note that an individual study can contain more than one grassland type), pastures and meadows (each n=26) and

various forms of heathland (n=10). Most of the pastures, meadows and natural grasslands were extensive mountainous or alpine grasslands. There were relatively few studies in intensive or improved permanent grasslands and wood-pastures (see also Table S4). Typically, regional studies did not indicate the specifics of the grasslands in focus, while plot scale studies gave detailed descriptions of the vegetation composition. Therefore, we could not compare threats according to grassland types.

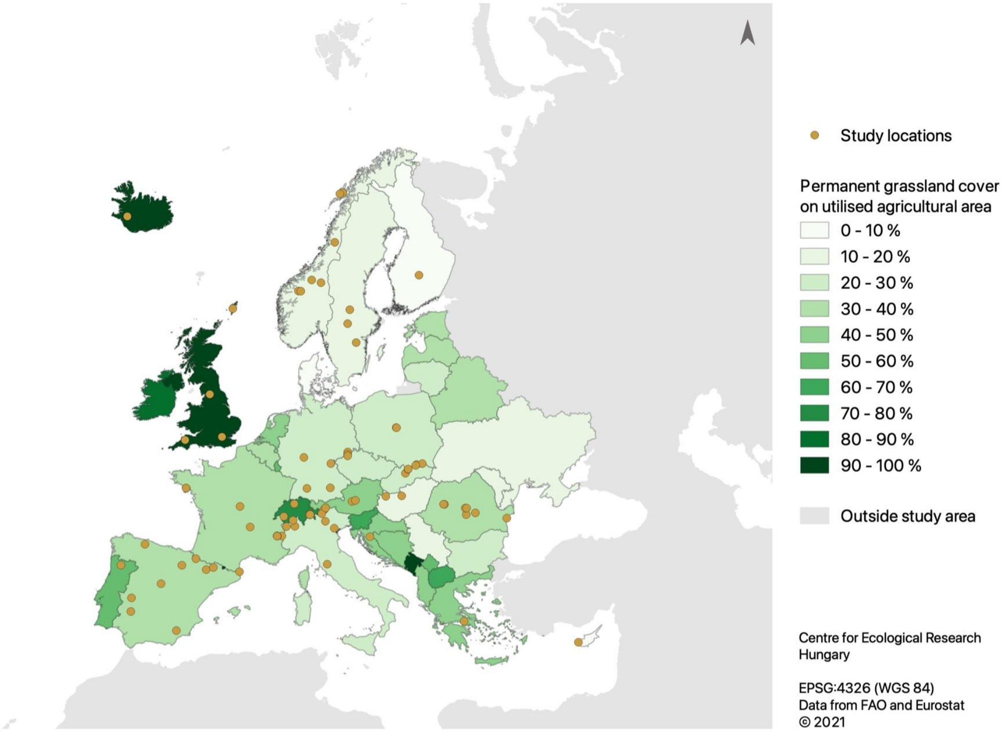

Figure S3. Study locations and percentage cover of permanent grassland on the utilised agricultural area in Europe. Note that several study locations per paper can occur. Data from Faostat (2021), except for Kosovo (Eurostat 2020).

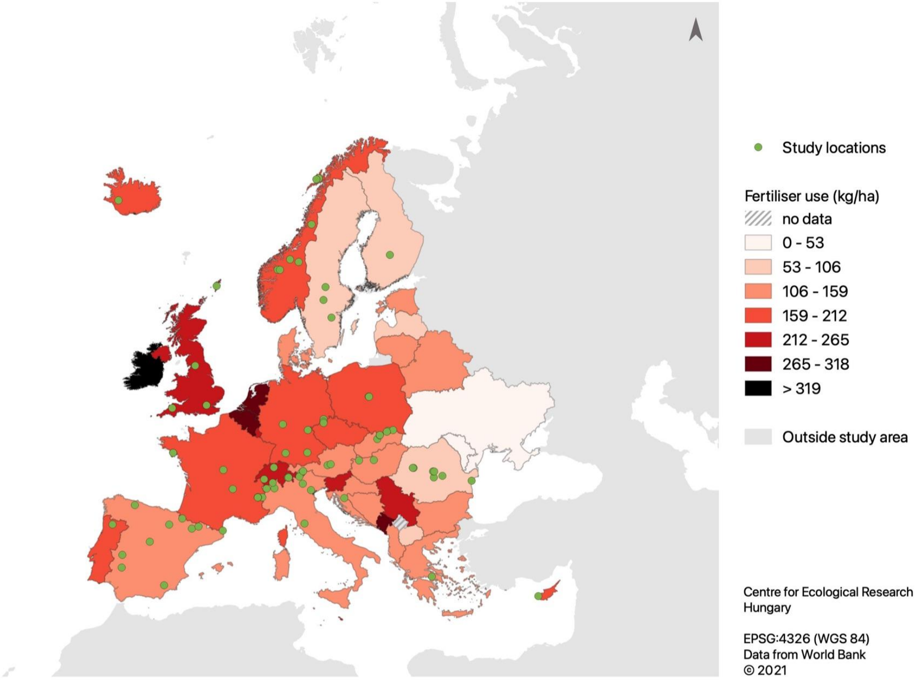

Figure S4. Study locations and fertiliser use (kg/ha) in Europe. Note that several study locations per paper can occur. Data from World Bank (2018).

Table S4. General characteristics of the 77 included studies.

	Characteristics		No. of studies
Study approach	Classical field studies		16
	Stakeholder surveys		
		Residents	7
		Tourists	7
		Farmers	9
		Others (experts, park managers etc.)	9
	Regional studies on sustainable development and tourism		12
	Economic valuation of landscape elements or scenarios		6
	General discourse of threats to a region		4
	Mapping study		3
	Ecosystem assessment		3
	Restoration study		1
Study scale	Regional-scale		54
	Plot scale		13
	Landscape-scale		9
	Country scale		1

Table S4. *continued*

Biogeographical region	Alpine	35
	Continental	14
	Atlantic	10
	Mediterranean	10
	Boreal	4
	Pannonian	2
	Arctic	1
	Black Sea	1
Country of study (number of study locations; corresponding superscripts indicate transboundary study locations that were not included as separate studies)	Spain	10
	France	7
	Italy	7[1]
	Norway	7
	Poland	7
	Romania	7
	Germany	6[2]
	Switzerland	6
	Austria	5[1]
	UK	4
	Sweden	3
	Czech Republic	2[2]
	Hungary	2
	Croatia	1
	Cyprus	1
	Finland	1
	Greece	1
	Iceland	1
	Portugal	1
	Slovakia	1
Grassland type (note that one study can contain several types)	Pasture	31
	Meadows	30
	Natural grasslands	18
	Heathland	10
	Improved (reseeded, fertilised)	6
	Wood-pasture	5

	Rangeland	4
	Peatland	2
	Orchard with meadow-pasture mix	2
	Moss-lichen vegetation	1
	Not specified	6

References

Eurostat. 2020. Share of main land types in utilised agricultural area (UAA) by NUTS 2 regions. Retrieved 9 September, 2020, from https://ec.europa.eu/eurostat/web/main/data/database.

Faostat. 2020. Land use indicators. Land under permanent meadows and pastures. Data from 2018. Retrieved 15 October, 2020, from https://www.fao.org/faostat/en/#data.

Gąsienica Byrcyn, W. 1992. The history and present role of the Tatra National Park. *Mountain Research & Development* 12: 205–210. doi:10.2307/3673791.

Gómez-Limón, F.J., and J.V. de Lucio. 1995. Recreational activities and loss of diversity in grasslands in Alta Manzanares Natural Park, Spain. *Biological Conservation* 74: 99–105. doi:10.1016/0006-3207(95)00018-Y.

Grabherr, G. 1982. The impact of trampling by tourists on a high altitudinal grassland in the Tyrolean Alps, Austria. *Vegetatio* 48: 209–217. doi:10.1007/BF00055262.

Schaminée, J.H.J., and M.H. Meertens. 1992. The influence of human activities on the vegetation of the subalpine zone of the Monts du Forez (Massif Central, France). *Preslia* 64: 327–342.

World Bank. 2018. Fertilizer consumption (kilograms per hectare of arable land). Retrieved 05 March, 2021, from https://data.worldbank.org/indicator/AG.CON.FERT.ZS?most_recent_year_desc=true

Wytrzens, H.K., and C. Mayer. 1999. Multiple use of alpine grassland in Austria and the implications for agricultural policy. *Bodenkultur* 50: 251–261.

Figure S5 (next page). Detailed representation of threats to cultural ecosystem services in permanent grasslands (see also Fig. 2 in the main text). The number of studies involved is mentioned for each category. Note that a study can contribute to more than one node if several aspects were mentioned.

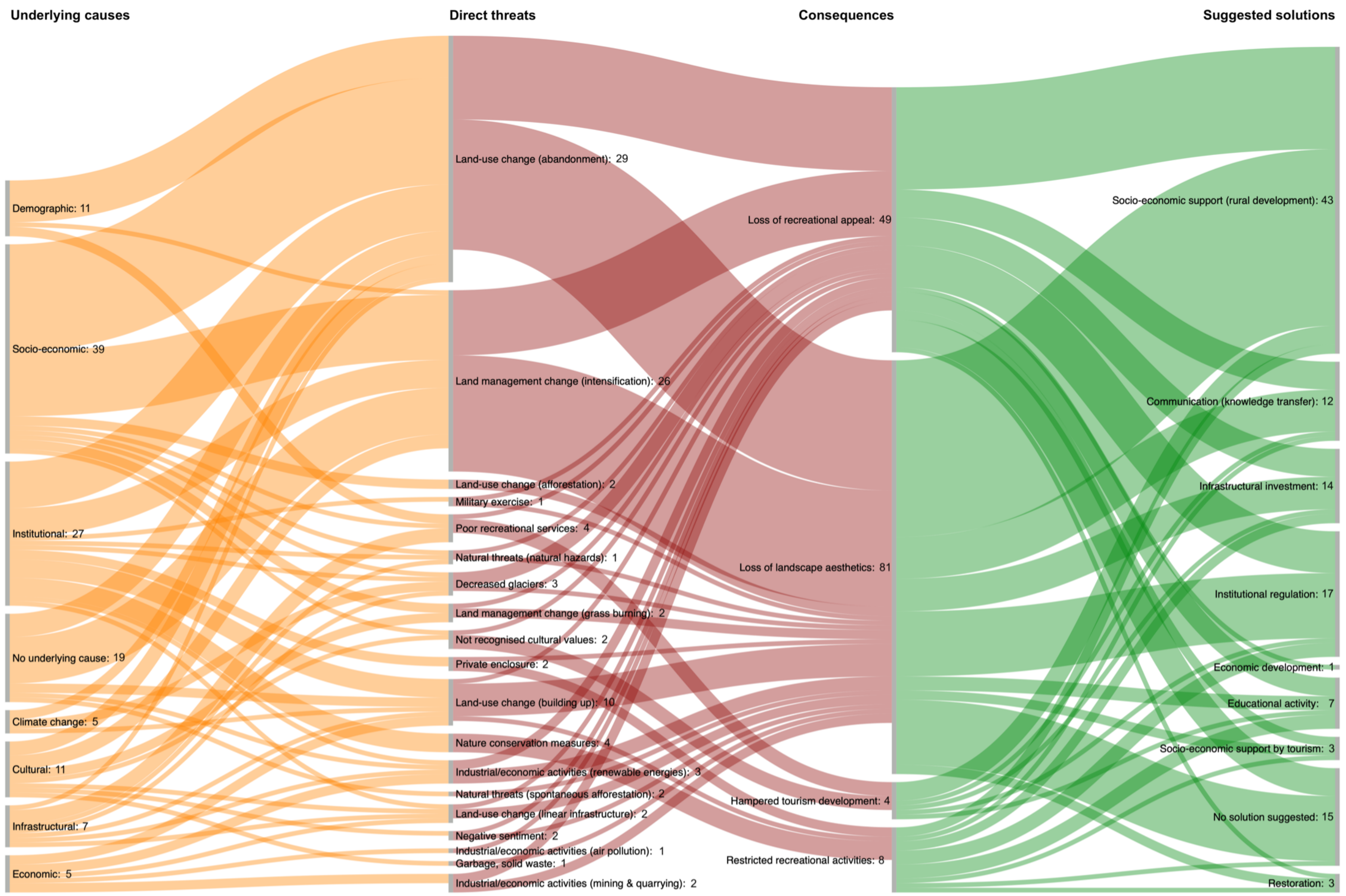

Underlying causes
Direct threats
Consequences
Suggested solutions
Demographic: 11
Socio-economic: 39
Institutional: 27
No underlying cause: 19
Climate change: 5
Cultural: 11
Infrastructural: 7
Economic: 5
Land-use change (abandonment): 29
Land management change (intensification): 26
Land-use change (afforestation): 2
Military exercise: 1
Poor recreational services: 4
Natural threats (natural hazards): 1
Decreased glaciers: 3
Land management change (grass burning): 2
Not recognised cultural values: 2
Private enclosure: 2
Land-use change (building up): 10
Nature conservation measures: 4
Industrial/economic activities (renewable energies): 3
Natural threats (spontaneous afforestation): 2
Land-use change (linear infrastructure): 2
Negative sentiment: 2
Industrial/economic activities (air pollution): 1
Garbage, solid waste: 1
Industrial/economic activities (mining & quarrying): 2
Loss of recreational appeal: 49
Loss of landscape aesthetics: 81
Hampered tourism development: 4
Restricted recreational activities: 8
Socio-economic support (rural development): 43
Communication (knowledge transfer): 12
Infrastructural investment: 14
Institutional regulation: 17
Economic development: 1
Educational activity: 7
Socio-economic support by tourism: 3
No solution suggested: 15
Restoration: 3

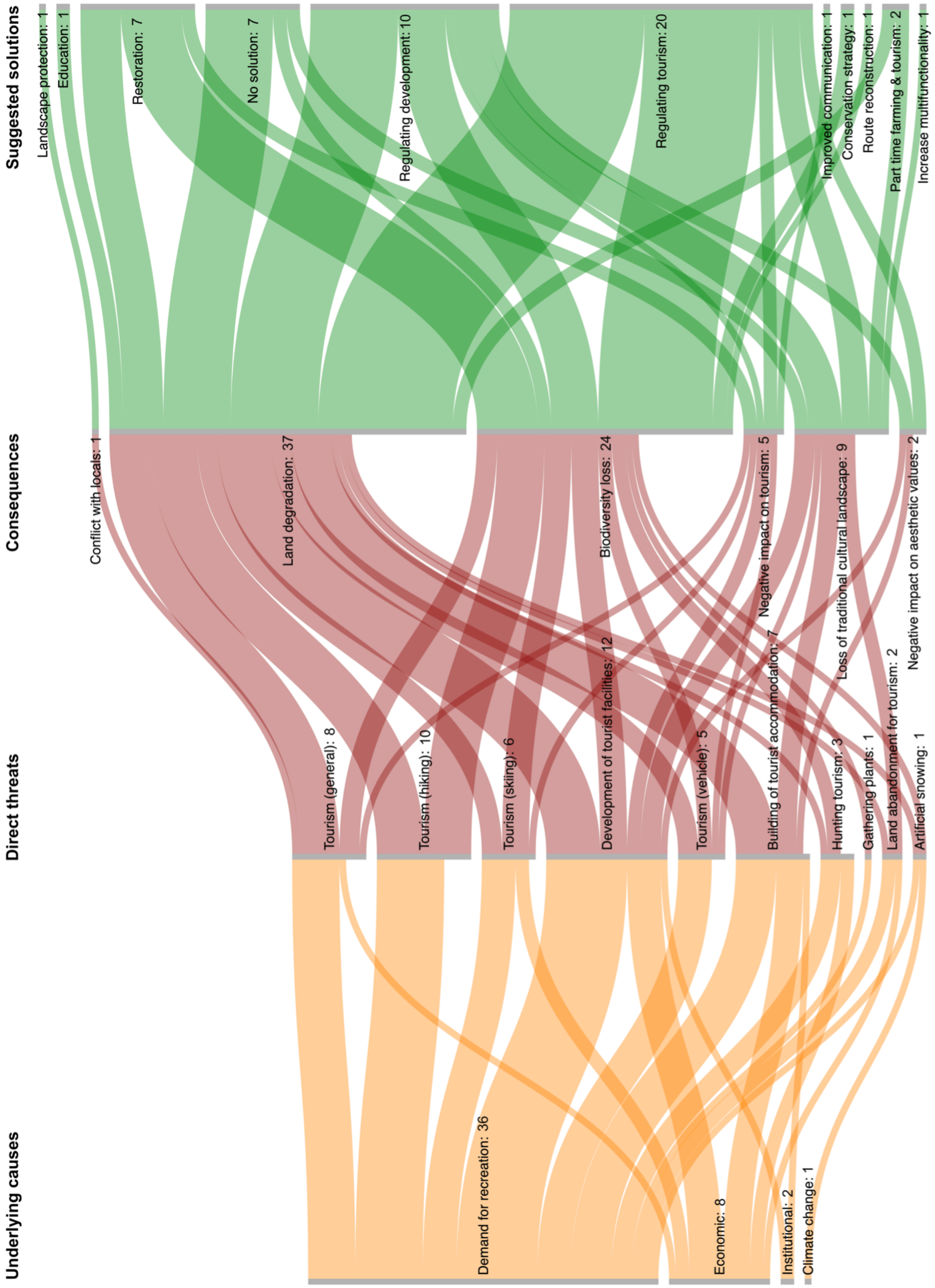

Figure S6. Detailed representation of cultural ecosystem services as threats to permanent grasslands (see also Fig. 3 in the main text). The number of studies involved is mentioned for each category. Note that a study can contribute to more than one node if several aspects were mentioned.
